# Loss of muscleblind splicing factor shortens *Caenorhabditis elegans* lifespan by reducing the activity of p38 MAPK/PMK-1 and transcription factors ATF-7 and Nrf/SKN-1

**DOI:** 10.1093/genetics/iyab114

**Published:** 2021-07-22

**Authors:** Olli Matilainen, Ana R S Ribeiro, Jens Verbeeren, Murat Cetinbas, Heini Sood, Ruslan I Sadreyev, Susana M D A Garcia

**Affiliations:** 1 Institute of Biotechnology, HiLIFE, University of Helsinki, Helsinki 00790, Finland; 2 Department of Molecular Biology, Massachusetts General Hospital and Harvard Medical School, Boston, MA 02114, USA

**Keywords:** Muscleblind splicing factor, *C. elegans*, lifespan, p38 MAPK, PMK-1, ATF-1, SKN-1, mitochondrial stress

## Abstract

Muscleblind-like splicing regulators (MBNLs) are RNA-binding factors that have an important role in developmental processes. Dysfunction of these factors is a key contributor of different neuromuscular degenerative disorders, including Myotonic Dystrophy type 1 (DM1). Since DM1 is a multisystemic disease characterized by symptoms resembling accelerated aging, we asked which cellular processes do MBNLs regulate that make them necessary for normal lifespan. By utilizing the model organism *Caenorhabditis elegans*, we found that loss of MBL-1 (the sole ortholog of mammalian MBNLs), which is known to be required for normal lifespan, shortens lifespan by decreasing the activity of p38 MAPK/PMK-1 as well as the function of transcription factors ATF-7 and SKN-1. Furthermore, we show that mitochondrial stress caused by the knockdown of mitochondrial electron transport chain components promotes the longevity of *mbl-1* mutants in a partially PMK-1-dependent manner. Together, the data establish a mechanism of how DM1-associated loss of muscleblind affects lifespan. Furthermore, this study suggests that mitochondrial stress could alleviate symptoms caused by the dysfunction of muscleblind splicing factor, creating a potential approach to investigate for therapy.

## Introduction

The human Muscleblind-like (MBNL) protein family consists of MBNL1, MBNL2, and MBNL3. As they regulate alternative splicing programs, polyadenylation, mRNA turnover, and localization (Pascual *et al.* 2006; Masuda *et al.* 2012; Wang *et al.* 2012; Batra *et al.* 2014; Xu *et al.* 2021), dysfunction of MBNLs has been found to be implicated in many degenerative diseases such as Huntington's disease‐like 2, spinocerebellar ataxia type 8, and polyglutamine disorders (Fernandez-Costa *et al.* 2011). However, MBNL dysfunction is best known for its role in myotonic dystrophies.

Myotonic dystrophy type 1 (DM1) and type 2 (DM2) are multisystemic diseases caused by 50-4000 CUG trinucleotide repeat expansions in the 3′ untranslated region (UTR) of the myotonic dystrophy protein kinase (*DMPK*) gene and 55–11,000 CCUG repeats in the first intron of the cellular nucleic acid-binding protein (*CNBP*) gene, respectively. DM1 is the most prevalent muscular dystrophy in adults, and its clinical features are more severe compared to DM2 (Meola and Cardani 2015). At the molecular level, repeat-containing RNAs form imperfect double-stranded structures that sequester, and thereby disrupt the function of MBNLs and several other RNA-binding factors (Miller *et al.* 2000; Fardaei *et al.* 2001), which leads to splicing defects (Ranum and Cooper 2006). Importantly, *Mbnl1* and *Mbnl2* knockout mice develop RNA splicing abnormalities that are characteristic of DM1 (Kanadia *et al.* 2003; Charizanis *et al.* 2012; Suenaga *et al.* 2012), demonstrating that the loss of MBNL splicing factors is a central contributor of DM1 pathogenesis. Since DM1 manifests with phenotypes commonly associated with aging (e.g. cataracts, muscular weakness, atrophy, insulin resistance, and metabolic dysfunction), it has also been considered as a disorder of accelerated aging (Mateos-Aierdi *et al.* 2015). Thus, the function of MBNL splicing factors may play an important regulatory role in aging.

MBNLs are well-conserved factors across animals. In *Caenorhabditis elegans*, MBL-1, the sole ortholog of mammalian MBNL proteins binds CUG and CCUG repeats (Sasagawa *et al.* 2009; Garcia *et al.* 2014) and is required for normal mRNA splicing (Norris *et al.* 2017; Thompson *et al.* 2019). MBL-1 appears to be required for normal muscle structure and function as well as for the proper formation of neuromuscular junction synapses and dendrite morphogenesis (Wang *et al.* 2008; Spilker *et al.* 2012; Antonacci *et al.* 2015), indicating that the loss of MBL-1 recapitulates DM1 patient phenotypes in *C. elegans*. Notably, it has been shown that the loss of MBL-1 shortens *C. elegans* lifespan (Wang *et al.* 2008; Sasagawa *et al.* 2009), but the mechanism behind this phenotype is not known.

Here we used *C. elegans* to elucidate the mechanism of how the loss of MBL-1 shortens lifespan. We found that MBL-1-deficient animals have reduced activity of the conserved p38 mitogen-activated protein kinase (MAPK) (PMK-1 in *C. elegans*) and its downstream transcription factors ATF-7 and SKN-1, which results in shortened lifespan. Furthermore, we demonstrate that mitochondrial stress activates a prolongevity response in *mbl-1* mutants which is partially p38 MAPK/PMK-1-dependent. These findings establish PMK-1, ATF-7, and SKN-1 as mechanistic links between the developmentally regulated muscleblind splicing factor and lifespan, and establish mitochondria as potential therapeutic targets to treat aging-associated symptoms of DM1.

## Materials and methods

### *Caenorhabditis elegans* strains and maintenance

For all experiments, *C. elegans* were maintained at 20°C on NGM plates (peptone, P4963, Merck; agar, A4550, Merck; NaCl, 746398, Merck). The N2 (Bristol) strain was used as the wild type. N2, *tir-1(qd4)* (ZD101), *nsy-1(ag3)* (AU3), *sek-1(km4)* (KU4), *pmk-1(km25)* (KU25), and *gst-4p::gfp* (CL2166) were obtained from the Caenorhabditis Genetics Center (CGC). *mbl-1(tm1563)* strain was received from National BioResource Project (NBRP), and outcrossed five times with N2. *mbl-1(tm1563)*; *gst-4p::gfp* (GAR132) and *pmk-1(km25)*; *mbl-1(tm1563)* (GAR139) crosses as well as extrachromosomal *mbl-1* overexpressing animals (GAR152: (*iceEx51*[*mbl-1p(1):: mbl-1-isoA::mCherry*] [*mbl-1p(2)::mbl-1-isoA::mCherry*] [*mbl-1p(1):: mbl-1- isoB::mCherry*] [*mbl-1p(2)::mbl-1-isoB::mCherry*] [*mbl-1p(1)::mbl-1-isoC::mCherry*] [*mbl-1p(2)::mbl-1-isoC::mCherry*])) were created within this study.

### *Pseudomonas aeruginosa* assay

3.5-cm NG plates were seeded with 40 µL of overnight grown *Pseudomonas aeruginosa* (PA14) suspension and incubated at 37°C for 24 h (Tan *et al.* 1999). Twenty microliters of SDS 2% was added to the edges of the plate to prevent the escape of the animals. *Caenorhabditis* *elegans* were transferred to PA14 plates at L4 stage and incubated at 25°C. Animals were scored daily for survival based on their ability to respond to touch. Animals dehydrated on the wall of the plate were censored from the analysis. For Western blot upon PA14 treatment, animals were transferred to PA14 plates at L4 stage and collected 24 h later as day 1 adults.

### RNA interference

EV refers to “Empty Vector,” which was used as a control in RNA interference (RNAi) experiments. *tir-1*, *pmk-1*, *atf-7*, *skn-1*, and *cco-1* RNAi clones were taken from either Ahringer or Vidal RNAi library. *daf-16* (#34833) and *daf-2* RNAi (#34834) clones were obtained from Addgene. *mbl-1* RNAi was cloned from *C. elegans* cDNA by using 5′-CTTGAGCTCTTCGACGAAAACAGTAATGCCGC-3′ and 5′-CTTCTCGAGCTAGAATGGTGGTGGCTGCATG-3′ primers. RNAi was performed using the feeding protocol as described earlier (Timmons *et al.* 2001). Lifespan experiments were initiated by letting gravid hermaphrodites (P0 generation) to lay eggs on RNAi/NGM plates, and P1 generation was scored for lifespan. In some lifespan experiments, animals were bleached and let to hatch overnight in M9 before plating P1 generation as L1 larvae to experimental plates. These two alternative ways to initiate lifespan did not affect the conclusions made from the experiments. For qRT-PCR and Western blot animals were bleached and let to hatch overnight in M9 before plating L1 larvae for experimental plates.

### Generation of *mbl-1* overexpression strains

GAR152 was generated by microinjection of six different transgenic constructs at 15 ng/μl concentration each. These transgenes were expressed as extrachromosomal arrays and express three different isoforms (*K02H8.1a*; isoform A, *K02H8.1b*; isoform B, and *K02H8.1c*, isoform C) fused to mCherry, under two different endogenous promoters; *mbl-1p(1)* and *mbl-1p(2)*. Constructs were made through modification of a plasmid expressing *mbl-1* isoform A fused to mCherry under the *unc-54* promoter (Garcia *et al.* 2014). PCR-amplified fragments from wild-type *C. elegans* genome, representing two endogenous *mbl-1* promoter regions, replaced the *unc-54* promoter. For promoter 1, *mbl-1p(1)*, primers 5′-TACGCATGCAGGCCCTATATATTCCATCTCAAT-3′, containing a *Sph*I site, and 5′-TACGGATCCTCTGAAAAGTAGGAAAAAGATTGGC, containing a *Bam*HI site were used. For promoter two, *mbl-1p(2)*, primers 5′‐AACTGCAGGTGCAATGGGCTACTGATCTCC‐3′ and 5′‐CGGGATCCCATTCCGTCACTTGCAAAGAAC‐3′ were used, containing *Pst*I and *Bam*HI sites, respectively. Plasmids expressing isoforms B and C were derived from these constructs through PCR using forward primers 5′‐CAGCTACAAACTGCCGC CT‐3′ (isoform B) and 5′-GGAGCTGTACCAATGAAGCGAC‐3′ (isoform C), and reverse primer 5′‐CTGATTCACTGCCGCTGCTGTATAAG‐3′.

### Western blot

Animals were collected at indicated age and frozen in liquid nitrogen. Animals were lysed in protease inhibitor cocktail (Abcam, #ab65621) and 3× phosphatase inhibitor (Thermo Scientific, #78420)-supplemented RIPA buffer (bioWORLD) with a micropestle in 1.5-ml Eppendorf tubes. Lysates were resolved on 4–15% precast polyacrylamide gels (Bio-Rad). Phospho-p38 MAPK (used with 1:1000 dilution) was purchased from Cell Signaling Technology (#4511S) and α-tubulin antibody (used with 1:5000 dilution) from Merck (T5168). Secondary antibodies Goat Anti-Rabbit IgG H&L (HRP) (ab97051) and Goat Anti-Mouse IgG H&L (HRP) (ab97023) (used with 1:10,000 dilution) were purchased from Abcam. SuperSignal™ West Pico PLUS Chemiluminescent Substrate (#34577, Thermo Fisher Scientific) was used for protein detection. Western blots were quantified by using Fiji (see Supplementary Table S6 for Western blot quantifications).

### RNA sequencing

N2 and *mbl-1(tm1563)* strains were synchronized by bleaching and plated as L1 larvae on RNAi plates seeded with HT115 bacteria carrying EV ( control vector for RNAi). To prevent the hatching of the progeny, animals were transferred to plates containing 10 μM of 5-fluorouracil (Sigma) at the L4 stage. Animals were collected on day 2 of adulthood (three biological replicates for both strains) and frozen in liquid nitrogen. Total RNA was extracted with TRIzol Reagent (Ambion) and assessed for degradation using Agilent 2100 Bioanalyzer. Illumina Truseq stranded polyA-mRNA library was prepared and sequenced for 86 cycles at the DNA Sequencing and Genomics Laboratory (Institute of Biotechnology, University of Helsinki). The six samples were multiplexed and sequenced on one lane of Illumina NextSeq 500, yielding circa 18–20 million reads per sample. Sequencing reads were mapped to the *C.* *elegans* reference transcriptome (WBcel235 assembly) using STAR (Dobin *et al.* 2013). Read counts over transcripts were calculated using HTSeq (Anders *et al.* 2015). For differential expression analysis, we used the edgeR method (Robinson *et al.* 2010) and classified genes as differentially expressed based on the cutoffs of twofold change in expression value and false discovery rates (FDR) below 0.05. For differential splicing analysis, we used the diffSpliceDGE function in the edgeR package. Splicing isoforms were detected by applying Simes-statistical method within diffSpliceDGE function with an FDR cutoff of 0.05.

### Lifespan analysis

*Caenorhabditis* *elegans* lifespan experiments were done at 20°C. At the L4 larval stage, animals were transferred to plates containing 10 µM of 5-Fluorouracil (Sigma) to prevent progeny production. Animals that had exploded vulva or that crawled off the plate were censored. Animals were counted as dead if there was no movement after poking with a platinum wire. Lifespans were checked every 1–3 days. For lifespan data, mean lifespan ± standard error (SE) is reported (see Supplementary Tables S1 and S2).

### Quantitative RT-PCR

Animals were collected at indicated age and frozen in liquid nitrogen. TRIzol Reagent (Ambion) was used to extract RNA. cDNA synthesis was done with QuantiTect Reverse Transcription Kit (Qiagen) and quantitative RT-PCR (qRT-PCR) reactions were run with the SYBR Green reagent (Roche) using Lightcycler 480 (Roche). qRT-PCR data were normalized to the expression of *cdc-42* and *pmp-3*. qRT-PCR oligos used in this study are provided in Supplementary Table S9. qRT-PCR experiments were performed with three biological replicates (see Supplementary Table S8 for raw qRT-PCR data) and repeated at least twice with similar results. Statistical significances were analyzed by using Student’s *t*-test or two-way ANOVA.

### Agarose gel-based splicing assay

Animals were collected at indicated age and frozen in liquid nitrogen. TRIzol Reagent (Ambion) was used to extract RNA. cDNA synthesis was done with QuantiTect Reverse Transcription Kit (Qiagen). Splicing of *unc-43* and *unc-104* mRNAs was analyzed by running PCR products in agarose gels. Oligos used for PCR are provided in Supplementary Table S10. Splicing assays were performed with three biological replicates. Agarose gels were quantified by using Fiji. Statistical significances were analyzed by using two-way ANOVA.

### Fluorescent imaging and oxidative stress assay

*gst-4p::GFP* reporter strains (wild-type and *mbl-1(tm1563)* background) were grown on EV RNAi plates and imaged as day 1 adults with a standard stereomicroscope with fluorescent light source (Zeiss). For induction of oxidative stress, 5% H_2_O_2_ (in H_2_O, H_2_O used as a control) was pipetted to EV plates containing 1-day adult *gst-4p::gfp* reporter animals. Worms were washed after 20 min, and let to recover on EV plates for 5 h before imaging. GFP fluorescence was quantified by using Fiji (see Supplementary Table S8 for GFP quantifications).

### Statistical analysis

Statistical analyses for qRT-PCR data were carried out in GraphPad Prism or Excel, and the data represent the mean of three biological replicates ± SD. Statistical analyses for Western blot data, splicing assays (agarose gels), and GFP fluorescence were carried out in GraphPad Prism. Statistical details can be found in the figures and figure legends. Statistical analyses for lifespan experiments were carried out in R by using the Cox proportional hazard regression. Statistical details for the lifespan data can be found in Supplementary Tables S1 and S2. Supplementary Table S1 shows calculations from combined independent lifespan experiments (from Supplementary Table S2).

### Data availability

Strains and plasmids are available upon request. The Gene Expression Omnibus (GEO) accession number for the RNA-seq data originating from this study is GSE146801.

Supplementary material is available at *GENETICS* online.

## Results

### Loss of MBL-1 leads to shortened lifespan and reduced expression of p38 MAPK/PMK-1-regulated genes

**Figure 1:**
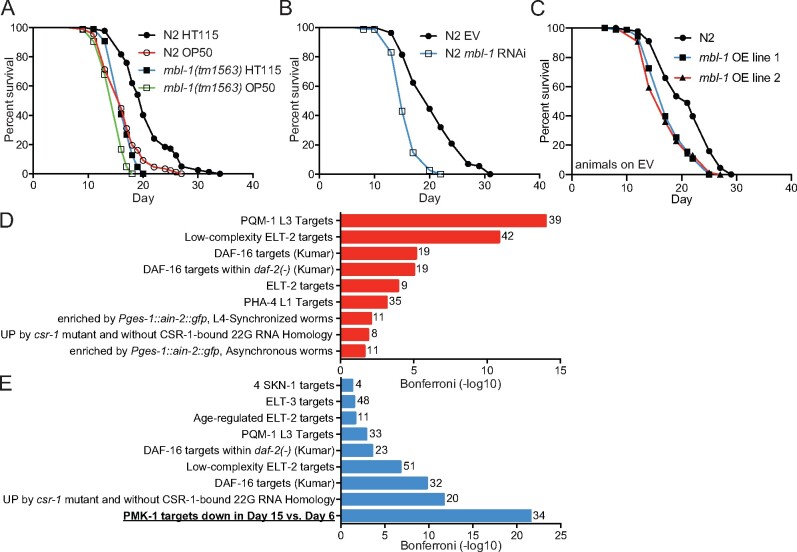
The loss of MBL-1 shortens lifespan and reduces the expression of p38 MAPK/PMK-1 target genes. (A) *mbl-1(tm1563)* mutants have shortened lifespan compared to wild type (N2) on both HT115 (*P* < 0.01) and OP50 (*P* < 0.01) *E. coli*. (B) *mbl-1* RNAi shortens lifespan compared to EV-treated N2 (*P* < 0.01). (C) Strains expressing extrachromosomal mCherry-tagged MBL-1 have short lifespan compared to N2 (*P* < 0.01). See Supplementary Tables S1 and S2 for lifespan statistics. (D) The most significant terms from WormExp output for upregulated genes (135) in day 2 adult *mbl-1(tm1563)* mutants compared to N2. (E) The most significant terms from WormExp output for downregulated genes (235) in day 2 adult *mbl-1(tm1563)* mutants compared to N2. In (D) and (E) numbers next to bars represent number of genes associated with the particular WormExp term. See Supplementary Tables S4 and S5 for differentially expressed genes between N2 and *mbl-1(tm1563)* mutants and WormExp statistics, respectively.

To examine how the loss of MBL-1 affects *C. elegans* lifespan, we utilized a strain carrying the *mbl-1(tm1563)* allele (513-bp deletion) that eliminates an exon shared among all *mbl-1* isoforms and creates a putative null allele. This deletion has been shown to shorten lifespan (Sasagawa *et al.* 2009). We repeated this experiment, performing lifespan assays on two *Escherichia* *coli* strains, HT115 and OP50, which are both widely used in *C. elegans* experiments. We found that *mbl-1(tm1563)* mutant animals have significantly shorter lifespan on both bacterial strains compared to wild-type *C. elegans* (N2) (Figure 1A and Supplementary Tables S1 and S2). Interestingly, loss of MBL-1 has a more drastic decrease in lifespan on HT115 compared to OP50. One potential explanation is that the microbiome could modulate lifespan partly through MBL-1. As many experiments in this study take advantage of RNAi, all following experiments (with the exception of the *P.* *aeruginosa* resistance assay) were performed using HT115 bacteria. After confirming the earlier finding that *mbl-1(tm1563)* mutants have shortened lifespan (Sasagawa *et al.* 2009) (Figure 1A and Supplementary Tables S1 and S2), we asked whether knockdown of *mbl-1* produces a similar phenotype. It has been shown previously that *mbl-1* RNAi shortens lifespan (Wang *et al.* 2008). Likewise, we also found that *mbl-1* RNAi shortens N2 lifespan compared to treatment with EV (control RNAi) (Figure 1B and Supplementary Tables S1 and S2), thus further demonstrating that disruptions to MBL-1 function affect lifespan. Interestingly, simultaneous overexpression of three mCherry-tagged *mbl-1* isoforms (Supplementary Figure S1, A and B) under endogenous *mbl-1* promoters also shortens lifespan compared to N2 (Figure 1C and Supplementary Tables S1 and S2), indicating that an optimal level of this developmentally regulated splicing factor is required for normal lifespan. Next, we asked whether MBL-1 activity decreases with aging. For this purpose, we examined the *unc-43* and *unc-104* genes, known targets of MBL-1-mediated splicing (Norris *et al.* 2017), for age-related changes in their alternative splicing patterns (Supplementary Figure S2A). PCR analysis revealed that MBL-1 is required for exon exclusion events in both *unc-43* and *unc-104* (Supplementary Fig S2, B–E and Table S3). Day 4 adult N2 animals show enrichment of longer *unc-43* isoform, which is observed as early as L4 larval stage with *mbl-1(tm1563)* mutants (Supplementary Figure S2, B and C and Table S3), whereas *unc-104* splicing shows a similar enrichment already in day 2 adult N2 (Supplementary Figure S2, D and E and Table S3). These data suggest that MBL-1 activity decreases upon aging.

Since the reduced activity of MBNLs is a hallmark of DM1, we focused on the mechanism by which the loss of MBL-1 shortens lifespan. As an initial step, we performed RNA-seq of day 2 adult (day 5 from hatch) N2 and *mbl-1(tm1563)* mutants. Our analysis revealed that 135 genes are up- and 235 downregulated (at least 2-fold change in expression, FDR < 0.05) in *mbl-1(tm1563)* mutants compared to N2 (Supplementary Table S4). In order to identify aging-associated signaling pathways linking the loss of MBL-1 with shortened lifespan, we utilized the WormExp database, which collates nearly all published *C. elegans* expression data sets from public databases (Yang *et al.* 2016). We found that genes differentially regulated in *mbl-1(tm1563)* mutants are modulated by factors such as PQM-1, ELT-2, DAF-16, and PMK-1 (Figure 1, D and E and Supplementary Table S5). Interestingly, PMK-1, which encodes the conserved p38 MAPK (p38 MAPK), was the only factor associated exclusively with downregulated genes (Figure 1E and Supplementary Table S5). More specifically, PMK-1-regulated genes, whose expression is reduced in day 15 vs. day 6 old *C. elegans* (Youngman *et al.* 2011), are enriched among the downregulated genes in *mbl-1(tm1563)* mutants (Figure 1E and Supplementary Table S5). Due to this enrichment, we focused on the link between p38 MAPK/PMK-1 and MBL-1 to regulate lifespan.

### Loss of MBL-1 does not impair innate immune response

**Figure 2:**
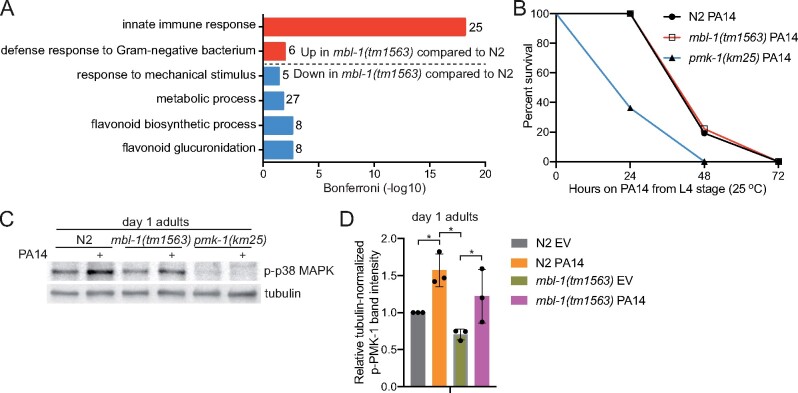
*mbl-1(tm1563)* mutants have innate immunity-associated gene expression signature, but normal response to *P. aeruginosa.* (A) GO terms (biological process) enriched among genes that are up- and downregulated in day 2 adult *mbl-1(tm1563)* mutants compared to N2 in RNA-seq (GSE146801). Numbers next to bars represent number of genes associated with the particular GO term. See Supplementary Table S4 for differentially expressed genes between N2 and *mbl-1(tm1563)* mutants. (B) *mbl-1(tm1563)* mutants have similar survival on pathogenic *P. aeruginosa* (PA14) compared to N2, but enhanced survival compared to *pmk-1(km25)* mutants. See Supplementary Tables S1 and S2 for lifespan statistics. (C) Both N2 and *mbl-1(tm1563)* mutants have significantly elevated level of phosphorylated p38 MAPK upon PA14 infection. Animals were transferred to PA14 at L4 stage and collected 24 h later (day 1 adults). (D) Quantified levels of phosphorylated p38 MAPK upon exposure to PA14. Bars represent the level of tubulin-normalized phosphorylated p38 MAPK relative to N2 EV with error bars indicating mean ± SD of three biological replicates (*P < 0.05, two-way ANOVA with Tukey’s test). See Supplementary Figure S3 for Western blot repeats and Supplementary Table S6 for Western blot quantifications.

When subjecting up- and downregulated genes to gene ontology (GO) analysis by using the DAVID database (Huang da *et al.* 2009), we found that innate immune response-related GO terms are enriched among genes upregulated in *mbl-1(tm1563)* mutants (Figure 2A). This is interesting since the WormExp analysis indicated that PMK-1, a central factor of innate immunity-regulating p38 MAPK signaling (Kim *et al.* 2002), has reduced activity in *mbl-1(tm1563)* mutants (Figure 1E and Supplementary Table S5). On the other hand, WormBase GO enrichment analysis of 34 PMK-1-regulated genes whose expression is decreased in *mbl-1(tm1563)* mutants (Figure 1E and Supplementary Table S5) reveals that only six genes are associated with innate immunity (Supplementary Table S5). These data indicate that the loss of MBL-1 does not impair the expression of PMK-1-regulated innate immunity genes. Nevertheless, we asked whether the loss of MBL-1 affects pathogen resistance, and for this experiment, we investigated the survival of *mbl-1(tm1563)* mutants on pathogenic *P.* *aeruginosa* (PA14) in a slow-killing assay. In this assay, we cultured PA14 on normal NGM plates where the killing occurs over the course of several days (Tan *et al.* 1999). *mbl-1(tm1563)* mutants were found to have similar survival on PA14 when compared to N2. This is in contrast to *pmk-1(km25)* mutants, which are extremely susceptible to PA14 (Figure 2B and Supplementary Tables S1 and S2). Furthermore, *mbl-1(tm1563)* mutants show significantly elevated levels of phosphorylated p38 MAPK/PMK-1 upon PA14 infection (Figure 2, C and D and Supplementary Figure S3 and Table S6). The data demonstrate that MBL-1 dysfunction does not impair the ability to mount a p38 MAPK/PMK-1-mediated innate immune response upon infection.

### *mbl-1(tm1563)* mutants have reduced level of activated p38 MAPK/PMK-1, which contributes to their short lifespan

**Figure 3:**
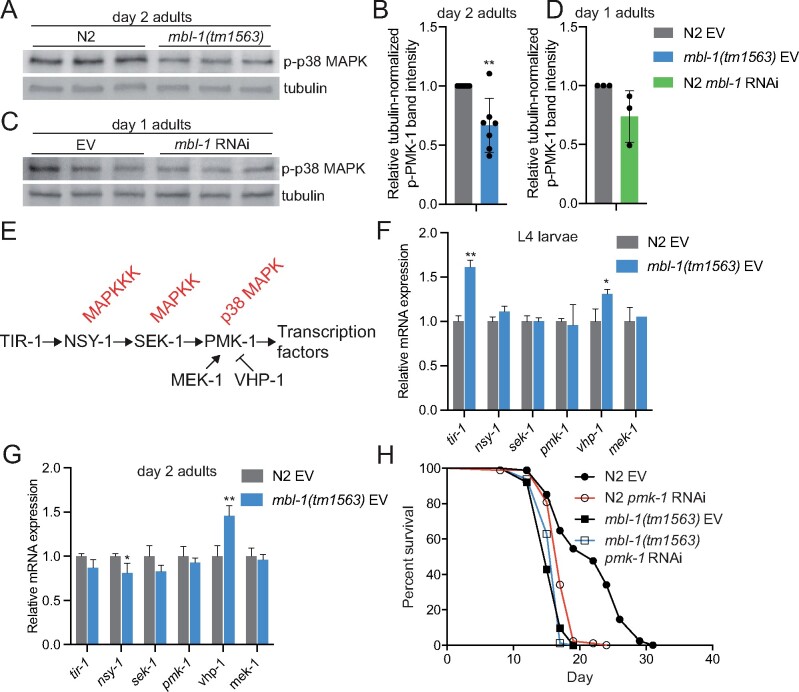
Decreased p38 MAPK/PMK-1 activation at nonpathogenic conditions shortens *mbl-1(tm1563)* mutant lifespan. (A) Day 2 adult *mbl-1(tm1563)* mutants have reduced level of phosphorylated p38 MAPK compared to N2. Shown are technical replicates of one biological replicate. (B) Quantified level of phosphorylated p38 MAPK in day 2 adult N2 and *mbl-1(tm1563)* mutants. (C) *mbl-1* RNAi does not significantly reduce the level of phosphorylated p38 MAPK in day 1 adult N2. (D) Quantified levels of phosphorylated p38 MAPK upon *mbl-1* RNAi in day 1 adult N2. In (B) and (D), bars represent the level of tubulin-normalized phosphorylated p38 MAPK relative to EV-treated N2 with error bars indicating mean ± SD of (B) seven and (D) three biological replicates (**P < 0.01, unpaired Student’s *t*-test). See Supplementary Figures S3 and S4 for Western blot repeats and Supplementary Table S6 for Western blot quantifications. (E) The *C. elegans* p38 MAPK pathway. (F) The expression of p38 MAPK pathway components in L4 larvae N2 and *mbl-1(tm1563)* mutants. (G) The expression of p38 MAPK pathway components in day 2 adult N2 and *mbl-1(tm1563)* mutants. In (F) and (G), bars represent mRNA levels relative to N2 EV with error bars indicating mean ± SD of three biological replicates, each with three technical replicates (*P < 0.05, **P < 0.01, unpaired Student’s *t*-test). See Supplementary Table S8 for raw qRT-PCR data. (H) *pmk-1* RNAi causes reduction in N2 lifespan (*P* < 0.01) but does not affect the lifespan of *mbl-1(tm1563)* mutants (*P* = 0.605). See Supplementary Tables S1 and S2 for lifespan statistics.

Since our RNA-seq data suggested that *mbl-1(tm1563)* mutants exhibit decreased PMK-1 activity (Figure 1E and Supplementary Table S5), we analyzed whether the level of phosphorylated p38 MAPK/PMK-1, which is traditionally used to assess the activity of this kinase, is altered in these mutants. Western blot analysis showed that day 2 old *mbl-1(tm1563)* mutants have a reduced level of phosphorylated PMK-1 (Figure 3, A and B, Supplementary Figures S3 and S4 and Table S6), thus demonstrating that basal p38 MAPK/PMK-1 activity is decreased upon disrupted MBL-1 function. *mbl-1* RNAi does not significantly affect PMK-1 phosphorylation (Figure 3, C and D, Supplementary Figure S3 and Table S6), which could be due to insufficient knockdown efficiency. However, the quantified mean of phosphorylated PMK-1 levels from three independent experiments (Figure 3C) indicates that, in addition to *mbl-1* mutation, *mbl-1* knockdown also modulates PMK-1 phosphorylation.

To examine whether MBL-1-regulated splicing is implicated in modulating the activity of p38 MAPK/PMK-1, we analyzed our RNA-seq data for alternative splicing (Supplementary Table S7). However, this analysis, or previously published investigation of MBL-1-dependent splicing in L4 larvae (Norris *et al.* 2017), did not reveal any obvious splicing event that could cause the decline in the activity of p38 MAPK/PMK-1 in *mbl-1* mutant animals. In addition to alternative splicing, MBNL1 regulates also mRNA stability (Masuda *et al.* 2012). Therefore, we asked whether mRNA levels of *C. elegans* p38 MAPK pathway (Figure 3E) genes are altered in *mbl-1(tm1563)* mutants. We found that in L4 larvae the expression of *tir-1* is upregulated in *mbl-1(tm1563)* mutants, whereas the expression of *nsy-1*, *sek-1*, and *pmk-1* is unchanged (Figure 3F and Supplementary Table S8). In addition to p38 MAPK pathway genes, we examined the expression of *mek-1* MAPK kinase and *vhp-1* MAPK phosphatase, which both regulate PMK-1 activity (Kim *et al.* 2004). Interestingly, whereas *mek-1* expression is unchanged in *mbl-1(tm1563)* mutants, we found that the expression of *vhp-1* is increased in these animals (Figure 3F and Supplementary Table S8). Additionally, we found that the expression of *nsy-1* and *vhp-1* is decreased and increased, respectively, in day 2 adult *mbl-1(tm1563)* mutants (Figure 3G andSupplementary Table S8). Since VHP-1 negatively regulates p38 MAPK/PMK-1phosphorylation (Kim *et al.* 2004), it is likely that the elevated expression of *vhp-1* contributes to the reduced p38 MAPK/PMK-1 activity in *mbl-1(tm1563)* mutants.

To determine whether the decreased p38 MAPK/PMK-1 activity contributes to the short lifespan of *mbl-1(tm1563)* mutants, we studied how these mutants are affected by the knockdown of *pmk-1.* RNAi of *pmk-1* reduces N2 lifespan but does not affect the lifespan of *mbl-1(tm1563)* mutants (Figure 3H and Supplementary Tables S1 and S2). Next, we tested whether the shortened lifespan upon loss of MBL-1 is affected by the depletion of other components of the p38 MAPK pathway (Figure 3E). We found that RNAi of *tir-1* shortens N2 lifespan but does not affect the lifespan of *mbl-1(tm1563)* mutants (Supplementary Figure S5A and Tables S1 and S2). Furthermore, both *nsy-1(ag3)* and *sek-1(km4)* mutants have shortened lifespans, which are not affected by *mbl-1* RNAi (Supplementary Figure S5B and Tables S1 and S2). These data indicate that the dysfunction of p38 MAPK signaling pathway contributes to the short lifespan of *mbl-1(tm1563)* mutants*.* We also examined how the knockdown of *mek-1* and *vhp-1* affects the lifespan of *mbl-1(tm1563)* mutants. Interestingly, we found that *mek-1* RNAi does not affect N2 lifespan, but further shortens the lifespan of *mbl-1(tm1563)* mutants (Supplementary Figure S5C and Tables S1 and S2). Since MEK-1 is part of the c-Jun N-terminal kinase (JNK)-signaling pathway (Koga *et al.* 2000), it is possible that JNK signaling compensates for the reduced p38 MAPK/PMK-1 activity in *mbl-1(tm1563)* mutants. Since *vhp-1* RNAi perturbs development when initiated at L1 stage, we transferred animals from control RNAi (EV) to *vhp-1* RNAi as day 1 adults. Unlike *mek-1* RNAi, the adult-onset *vhp-1* knockdown shortens both N2 and *mbl-1(tm1563)* mutant lifespan (Supplementary Figure S5D and Tables S1 and S2). Since the loss of VHP-1 activates p38 MAPK/PMK-1 (Kim *et al.* 2004), our data support an earlier findings demonstrating that aberrant p38 MAPK/PMK-1 activation is toxic (Cheesman *et al.* 2016).

### Reduced activity of ATF-7 and SKN-1 transcription factors modulate *mbl-1(tm1563)* mutant lifespan

**Figure 4:**
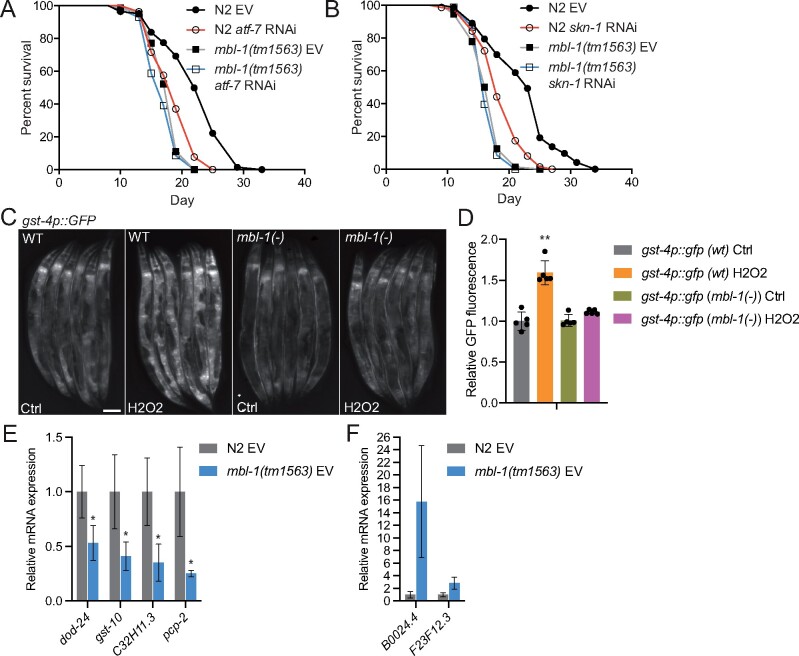
The short lifespan of *mbl-1(tm1563)* mutants is due to the dysfunction of ATF-7 and SKN-1 transcription factors. (A) *atf-7* RNAi shortens both N2 and *mbl-1(tm1563)* mutant lifespan (*P* < 0.01, *P* = 0.0291, respectively). (B) *skn-1* RNAi shortens both N2 and *mbl-1(tm1563)* mutant lifespan (*P* < 0.01, *P* = 0.0151, respectively). See Supplementary Tables S1 and S2 for lifespan statistics. (C) *mbl-1(tm1563)* mutants crossed with the transcriptional *gst-4p::GFP* reporter strain (CL2166) show reduced fluorescence upon acute oxidative stress. Scale bar, 100 μm. (D) Quantification of *gst-4p::GFP* reporter strain fluorescence. Quantification was done in groups of six animals, *n* = 30 animals for each condition. Bars represent GFP fluorescence relative to wild-type (wt) Ctrl with error bars indicating mean ± SD of five replicates (*P < 0.05, one-way ANOVA with Tukey’s test). (E and F) Expression of SKN-1-regulated genes in N2 and *mbl-1(tm1563)* mutants. Animals were grown on EV and collected for qRT-PCR analysis at day 2 adult stage. Bars represent mRNA levels relative to N2 with error bars indicating mean ± SD of three biological replicates, each with three technical replicates (*P < 0.05, unpaired Student’s *t*-test). See Supplementary Table S8 for raw qRT-PCR data.

In mammals, p38 MAPK signaling modulates cellular processes such as inflammation, development, cell differentiation, and senescence by controlling the activity of multiple transcriptional regulators (Zarubin and Han 2005). Therefore, it is likely that reduced activity of p38 MAPK/PMK-1 kinase shortens the lifespan of *mbl-1(tm1563)* mutants by affecting the function of transcription factors. To this end, we investigated how the downregulation of known PMK-1-regulated transcriptional regulators affect the lifespan of *mbl-1(tm1563)* mutants. Knockdown of *atf-7*, which encodes a transcription factor phosphorylated by PMK-1 in innate immune response (Shivers *et al.* 2010), induces larger decrease in N2 lifespan (14%) than in *mbl-1(tm1563)* mutant lifespan (5%) (Figure 4A and Supplementary Tables S1 and S2). This finding is further supported by Cox proportional hazard regression analysis, which shows that *atf-7* RNAi increases hazard ratio (HR) more in N2 background than in *mbl-1(tm1563)* background [HR compared to N2 EV: N2 *atf-7* RNAi 2.290, *mbl-1(tm1563)* EV 3.728, *mbl-1(tm1563) atf-7* RNAi 4.742; HR compared to *mbl-1(tm1563)* EV: *mbl-1(tm1563) atf-7* RNAi 1.272]. Since PMK-1 has been shown to phosphorylate and activate also SKN-1 (Inoue *et al.* 2005), we asked whether the aberrant function of this mammalian Nrf ortholog is linked with the shortened lifespan of *mbl-1(tm1563)* mutants. Similarly to *atf-7* knockdown, *skn-1* RNAi causes 1.8% decrease in *mbl-1(tm1563)* mutant lifespan, whereas it shortens N2 lifespan by 14% [HR compared to N2 EV: N2 *skn-1* RNAi 3.465, *mbl-1(tm1563)* EV 4.857, *mbl-1(tm1563) skn-1* RNAi 6.397; HR compared to *mbl-1(tm1563)* EV: *mbl-1(tm1563) skn-1* RNAi 1.317] (Figure 4B and Supplementary Tables S1 and S2). To study further whether the loss of MBL-1 modulates SKN-1 function, we crossed *mbl-1(tm1563)* mutants with the transcriptional reporter of *gst-4* (*gst-4p::gfp*), which is a well-known target of SKN-1 (Tullet *et al.* 2008). The *mbl-1(tm1563)* mutants were found to have impaired induction of *gst-4p::gfp* expression upon oxidative stress (5% H_2_O_2_ for 20 min, imaging after 5 h recovery) (Figure 4, C and D and Supplementary Table S8). Despite the requirement of MBL-1 on *gst-4* expression upon H_2_O_2_ treatment, MBL-1 expression pattern and level are not affected by oxidative stress (Supplementary Figure S1B). Additionally, our qRT-PCR analysis showed that the expression of *dod-24*, *gst-10*, *C32H11.3*, and *pcp-2*, whose expression is promoted by SKN-1 (Oliveira *et al.* 2009), is reduced in *mbl-1(tm1563)* mutants (Figure 4E), whereas the expression of *B0024.4* and *F23F12.3*, whose expression is negatively regulated by SKN-1 (Oliveira *et al.* 2009), is increased in *mbl-1(tm1563)* mutants (Figure 4F). Together, these data demonstrate that the function of SKN-1 is impaired in *mbl-1(tm1563)* mutants.

The expression of *gst-4* is regulated also by DAF-16 (Tullet *et al.* 2008), the sole FOXO ortholog in *C. elegans*. DAF-16 is regulated by insulin/IGF-1-like signaling, which is a well-known pathway affecting aging (Kenyon 2010). In addition to *gst-4*, our RNA-seq revealed that the expression of a subset of DAF-16/FOXO target genes is altered in *mbl-1(tm1563)* mutants (Figure 1, D and E and Supplementary Table S4). To further examine this, we analyzed mRNA levels of a few DAF-16 target genes by qRT-PCR in *mbl-1(tm1563)* mutants. We found that the expression of *sod-3* and *mtl-1*, which is promoted by DAF-16 (Murphy *et al.* 2003), is downregulated in *mbl-1(tm1563)* mutants, whereas the expression of *ins-7*, which is suppressed by DAF-16 (Murphy *et al.* 2003), is upregulated upon the loss of MBL-1 (Supplementary Figure S6A). Despite these changes in DAF-16 target gene expression, longevity assays showed that *daf-16* RNAi shortens N2 and *mbl-1(tm1563)* mutant lifespan by 6.8% and 9%, respectively (Supplementary Figure S6B and Tables S1 and S2), consistent with the notion that the loss of MBL-1 shortens lifespan independently of DAF-16. Additionally, RNAi of *daf-2*, which leads to increased longevity in an DAF-16-dependent manner (Kenyon *et al.* 1993), results in a similar increase in both N2 and *mbl-1(tm1563)* mutant lifespan (31.9% and 32.7%, respectively) (Supplementary Figure S6C and Tables S1 and S2). Taken together, these data indicate that loss of MBL-1 leads to shortened lifespan by reducing the activity of p38 MAPK/PMK-1, which in turn decreases the activity of ATF-7 and SKN-1 transcription factors. Furthermore, although the loss of MBL-1 causes differential expression of some DAF-16 target genes, the altered DAF-16 function does not contribute to the short lifespan of *mbl-1(tm1563)* mutants.

### Mitochondrial stress rescues the short lifespan of *mbl-1(tm1563)* mutants

Next, we asked whether targeting known aging mechanisms could modulate p38 MAPK activity, and thereby, rescue the short lifespan of *mbl-1(tm1563)* mutants. Interestingly, it has been shown that inhibition of mitochondrial respiratory chain complex I activates the PMK-1/ATF-7 signaling pathway (Chikka *et al.* 2016). Moreover, mitochondrial chaperone HSP-60 binds and stabilizes SEK-1 MAPK (see Figure 3E for *C. elegans* p38 MAPK pathway), thus promoting PMK-1 activation (Jeong *et al.* 2017). Due to these reports, we asked whether mitochondrial stress could extend *mbl-1(tm1563)* mutant lifespan.

**Figure 5:**
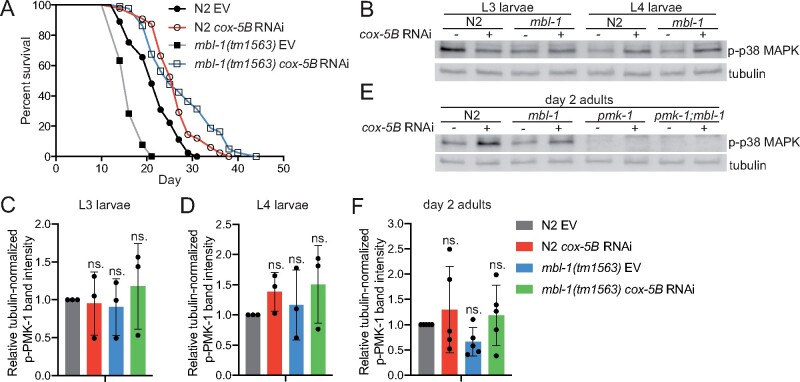
*cox-5B* RNAi-mediated mitochondrial stress modulates the level of phosphorylated p38 MAPK/PMK-1 and leads to increase in *mbl-1(tm1563)* mutant lifespan. (A) *cox-5B* RNAi-treated *mbl-1(tm1563)* mutants have longer lifespan than *cox-5B* RNAi-treated N2 animals (*P* < 0.01). (B) Western blot of phosphorylated p38 MAPK/PMK-1 in L3 and L4 larvae N2 and *mbl-1(tm1563)* mutants upon *cox-5B* RNAi. (C and D) Quantified levels of phosphorylated p38 MAPK upon *cox-5B* RNAi in (C) L3 and (D) L4 larvae N2 and *mbl-1(tm1563)* mutants. (E) Western blot of phosphorylated p38 MAPK/PMK-1 in day 2 adult N2 and *mbl-1(tm1563)* mutants upon *cox-5B* RNAi. (F) Quantified levels of phosphorylated p38 MAPK upon *cox-5B* RNAi in day 2 adult N2 and *mbl-1(tm1563)* mutants. In (C), (D), and (F) bars represent the level of tubulin-normalized phosphorylated p38 MAPK relative to EV-treated N2 with error bars indicating mean ± SD of (C-D) three and (F) five biological replicates (two-way ANOVA with Tukey’s test). See Supplementary Figure S4 for Western blot repeats and Supplementary Table S6 for Western blot quantifications.

To study a role for mitochondrial stress on the *mbl-1(tm1563)* mutant lifespan, we used RNAi of mitochondrial respiratory chain complex IV subunit *cox-5B* (also known as *cco-1*), which is known to extend lifespan (Dillin *et al.* 2002). As *cox-5B* RNAi also promotes innate immunity (Matilainen *et al.* 2017), we hypothesized that it leads to activation of p38 MAPK/PMK-1 kinase. Strikingly, we found that *cox-5B* RNAi leads to a significantly larger (*P* < 0.01, Cox proportional hazard regression analysis) lifespan extension in *mbl-1(tm1563)* mutants (+31%) compared to N2 animals (+15.3%) [HR compared to N2 EV: N2 *cox-5B* RNAi 0.42, *mbl-1(tm1563)* EV 3.426, *mbl-1(tm1563) cox-5B* RNAi 0.302; HR compared to *mbl-1(tm1563)* EV: *mbl-1(tm1563) cox-5B* RNAi 0.088] (Figure 5A and Supplementary Tables S1 and S2). To test whether *cox-5B* RNAi affects the p38 MAPK pathway, we performed Western blot to analyze p38 MAPK/PMK-1 phosphorylation. We found that *cox-5B* knockdown does not increase the level of phosphorylated p38 MAPK/PMK-1 in L3 larvae (Figure 5, B and C and Supplementary Figure S4 and Table S6). At L4 larval stage, *cox-5B* RNAi increases the level of phosphorylated p38 MAPK/PMK-1 in both N2 and *mbl-1(tm1563)* mutants in all individual experiments (Figure 5B and Supplementary Figure S4 and Table S6), but due to the variation between experiments, the increase is not statistically significant (Figure 5D). Also in day 2 adults, the effect of *cox-5B* RNAi on p38 MAPK/PMK-1 phosphorylation varies between experiments, and the increase in p38 MAPK/PMK-1 phosphorylation is not statistically significant (Figure 5, E and F and Supplementary Figure S4 and Table S6). However, in day 2 adult, *mbl-1(tm1563)* mutants *cox-5B* RNAi increases the level of phosphorylated p38 MAPK/PMK-1 in four out of five independent experiments (Supplementary Figure S4 and Table S6), thus indicating that similar to L4 larvae, *cox-5B* RNAi may modulate the activation p38 MAPK/PMK-1 in day 2 adults. Interestingly, it has been shown previously that RNAi of mitochondrial respiratory chain complex IV subunit *cox-4* (W09C5.8) does not promote the phosphorylation of p38 MAPK/PMK-1 (Chikka *et al.* 2016). Therefore, it is possible that knockdown of some, but not all, complex IV subunits activate p38 MAPK/PMK-1, which could be due to their role in complex IV function. Additionally, RNAi of separate complex IV subunits may lead to different knockdown efficiencies, which may also explain the divergence between our observations and the previously published data (Chikka *et al.* 2016).

**Figure 6:**
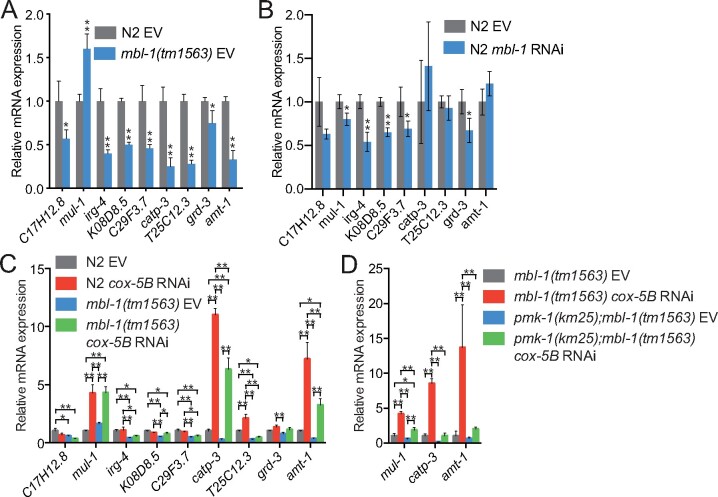
*cox-5B* RNAi-mediated mitochondrial stress modulates the expression of p38 MAPK/PMK-1 target genes. (A) The expression of PMK-1-regulated genes in L4 larvae N2 and *mbl-1(tm1563)* mutants. (B) The expression of PMK-1-regulated genes in *mbl-1* RNAi-treated L4 larvae N2. In (A) and (B), bars represent mRNA levels relative to N2 EV with error bars indicating mean ± SD of three biological replicates, each with three technical replicates (*P < 0.05, **P < 0.01, unpaired Student’s *t*-test). (C) The expression of PMK-1 regulated genes in L4 larvae N2 and *mbl-1(tm1563)* mutants upon *cox-5B* RNAi. The EV bars are from the same experiment presented in (A). (D) The expression of selected PMK-1 targets in L4 larvae *mbl-1(tm1563)* mutants and *pmk-1(km25); mbl-1(tm1563)* double mutants. In (C) and (D), bars represent mRNA levels relative to (C) N2 EV or (D) *mbl-1(tm1563)* EV with error bars indicating mean ± SD of three biological replicates, each with three technical replicates (*P < 0.05, **P < 0.01, two-way ANOVA with Tukey’s test). See Supplementary Table S8 for raw qRT-PCR data.

Next, we asked how *cox-5B* RNAi-mediated mitochondrial stress affects the expression of PMK-1-regulated genes (Troemel *et al.* 2006; Youngman *et al.* 2011). Importantly, the majority of PMK-1 target genes have decreased expression in *mbl-1(tm1563)* mutants (Figure 6A). Downregulated genes include *C17H12.8* and *K08D8.5* (Figure 6A), which are also regulated by ATF-7 (Shivers *et al.* 2010), providing further evidence for ATF-7 dysfunction in *mbl-1(tm1563)* mutants. Although less efficiently than *mbl-1(tm1563)* mutation, *mbl-1* RNAi also leads to decreased expression of a subset of PMK-1 target genes (Figure 6B). We found that *cox-5B* knockdown increases the expression of *mul-1*, *catp-3*, and *amt-1* in both N2 and *mbl-1(tm1563)* mutants (Figure 6C). To examine whether the upregulation of these genes is dependent on PMK-1, we compared their expression between *mbl-1(tm1563)* mutants and *pmk-1(km25)*; *mbl-1(tm1563)* double mutants. Loss of PMK-1 blunts the *cox-5B* RNAi-mediated induction of these genes (Figure 6D). The data further demonstrate that *cox-5B* knockdown modulates p38 MAPK/PMK-1 activity.

### *mbl-1(tm1563)* mutants require p38 MAPK/PMK-1 for maximal lifespan extension upon mitochondrial stress

**Figure 7:**
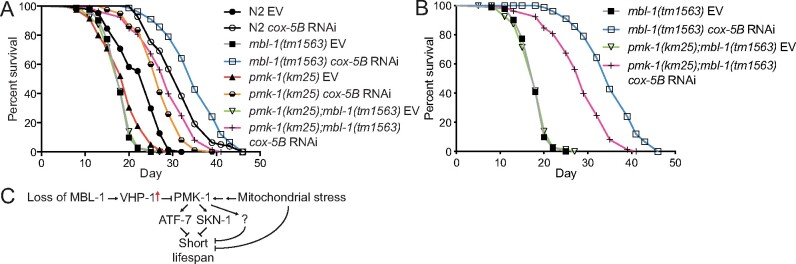
*cox-5B* RNAi-mediated mitochondrial stress results in PMK-1-dependent increase in *mbl-1(tm1563)* mutant lifespan. (A and B) *cox-5B* RNAi-treated *mbl-1(tm1563)* mutants have extended longevity compared to *cox-5B* RNAi-treated N2 (*P* < 0.01), *pmk-1(km25)* (*P* < 0.01) and *pmk-1(km25); mbl-1(tm1563)* (*P* < 0.01) mutants. Panel (B) shows the subset of lifespan curves presented in (A). See Supplementary Tables S1 and S2 for lifespan statistics. (C) Model based on the data presented here.

Although mitochondrial stress induces innate immune response (Pellegrino *et al.* 2014; Chikka *et al.* 2016; Jeong *et al.* 2017; Matilainen *et al.* 2017), p38 MAPK/PMK-1 has not been associated with mitochondria-mediated longevity. We asked whether PMK-1 is required for the increased *mbl-1(tm1563)* mutant lifespan upon *cox-5B* RNAi. Although *cox-5B* RNAi extends the lifespan of all tested strains (Figure 7, A and B and Supplementary Tables S1 and S2), *pmk-1(km25)*; *mbl-1(tm1563)* double mutation blocks the maximal lifespan extension observed with *cox-5B* RNAi-treated *mbl-1(tm1563)* mutants (Figure 7, A and B and Supplementary Tables S1 and S2). Importantly, *mbl-1(tm1563)* mutants and *pmk-1(km25)*; *mbl-1(tm1563)* double mutants have similar lifespan on control RNAi (EV) (Figure 7, A and B and Supplementary Tables S1 and S2). In addition to *cox-5B* RNAi, we also investigated whether PMK-1 is required for the lifespan increase of *mbl-1(tm1563)* mutants upon RNAi of mitochondrial respiratory chain complex I subunit *nduf-6*, which has been shown to increase p38 MAPK/PMK-1 phosphorylation (Chikka *et al.* 2016). Although on *nduf-6* RNAi *mbl-1(tm1563)* mutant lifespan is not increased beyond *nduf-6* RNAi-treated N2 animals, *nduf-6* knockdown leads to 30.6% increase in *mbl-1(tm1563)* mutant lifespan [HR compared to *mbl-1(tm1563)* EV: 0.089], whereas N2 lifespan is increased by 15.7% (HR compared to N2 EV: 0.507) (Supplementary Figure S7, A and B and Tables S1 and S2). Furthermore, similar to *cox-5B* RNAi, *pmk-1(km25)*; *mbl-1(tm1563)* double mutation blocks the maximal lifespan extension observed with *nduf-6* RNAi-treated *mbl-1(tm1563)* mutants (Supplementary Figure S7, A and B and Tables S1 and S2). Altogether, these data demonstrate that mitochondrial stress modulates the activity of p38 MAPK/PMK-1, thereby resulting in extended lifespan.

## Discussion

The data presented here support the hypothesis that loss of MBL-1 disrupts the mechanism maintaining basal p38 MAPK/PMK-1 activity in normal conditions, which leads to reduced activity of transcription factors ATF-7 and SKN-1 and shortened lifespan (Figure 7C). Since the decline in basal p38 MAPK/PMK-1 activity is an aging-associated process (Youngman *et al.* 2011), it can be hypothesized that the aberrant p38 MAPK signaling contributes to the premature aging-phenotype observed with MBNL deficiency in DM1 patients. Although *mbl-1(tm1563)* mutants have a decreased level of activated p38 MAPK/PMK-1 at nonpathogenic conditions, they do not show increased susceptibility to pathogens (Figure 2B and Supplementary Tables S1 and S2). This demonstrates that p38 MAPK/PMK-1 regulates lifespan independently of its role in innate immunity. In this context, chronic stress caused by aging differs from acute stress caused by pathogen infection. Therefore, we propose that moderate reduction in p38 MAPK/PMK-1 activity shortens *mbl-1(tm1563)* mutant lifespan, but it retains sufficient activity to respond to acute pathogenic bacterial stress. On the other hand, upregulation of innate immunity-associated genes in *mbl-1(tm1563)* mutants (Figure 2A) suggests that other pathogen surveillance-mechanisms may compensate the reduced p38 MAPK/PMK-1 activity, thus ensuring the normal survival of *mbl-1(tm1563)* mutants on PA14 (Figure 2B).

Importantly, the role of p38 MAPK/PMK-1 as a regulator of lifespan under nonpathogenic conditions is controversial, as it has been shown that *pmk-1(km25)* mutants have either normal lifespan (Kim *et al.* 2002; Troemel *et al.* 2006; Wu *et al.* 2019) or reduced lifespan (Pujol *et al.* 2008; Park *et al.* 2018; Zhou *et al.* 2019). Since PMK-1 is required for response to biotic stimulus, one possible explanation for differences between laboratories could be due to bacterial growth conditions, which in turn may be affected by brands of peptone and agar used for plate media. In this context, since *C. elegans* lifespan is regulated by their adaptive capacity to different diets (Pang and Curran 2014), lifespan analyses performed on HT115 and OP50 may result in different outcomes. Interestingly, although we found that both *pmk-1(km25)* mutants and *pmk-1* RNAi-treated animals have shortened lifespans, the HR is bigger for *pmk-1* RNAi-treated animals in Figure 3H (HR compared to N2 EV: 5.062) than for *pmk-1(km25)* mutants in Figure 7A (HR compared to N2 EV: 2.406) and in Supplementary Figure S7A (HR compared to N2 EV: 2.769). Although conclusions should not be made by comparing independent lifespan experiments, these data suggest that acute loss of PMK-1 could be more detrimental relative to chronic PMK-1 depletion.

Although demonstrating that the MBL-1 alternative splicing factor is required to maintain the basal p38 MAPK/PMK-1 activity and normal lifespan, this study leaves an open question about the molecular mechanism behind this phenotype. However, our data support that increased *vhp-1* expression (Figure 3, F and G andSupplementary Table S8) contributes to the reduced p38 MAPK/PMK-1 activity in *mbl-1(tm1563)* mutants. Interestingly, in addition to alternative splicing, MBNL also regulates mRNA stability (Masuda *et al.* 2012; Xu *et al.* 2021). Hence, it is possible that MBL-1 affects the activity of p38 MAPK/PMK-1 by promoting *vhp-1* mRNA decay (Figure 7C). Furthermore, p38 MAPK can also be activated by mechanical stimuli (Nguyen *et al.* 2000; Zhang *et al.* 2000; Sawada *et al.* 2001; Wang *et al.* 2005; Lewthwaite *et al.* 2006; Chaudhuri and Smith 2008; Dolinay *et al.* 2008; Hoffman *et al.* 2017). Since *mbl-1* mutants display the improper formation of neuromuscular junction synapses and altered locomotion (Spilker *et al.* 2012), one intriguing hypothesis is that dysfunctional neuromuscular communication and the consequent abnormal muscle function leads to lower level of mechanical stimuli, and thus to decreased basal p38 MAPK/PMK-1 activity. In this context, one interesting experiment would be to address whether electrical stimulation promotes basal p38 MAPK/PMK-1 activity and increases *mbl-1* mutant lifespan.

In addition to the model that MBL-1 promotes normal lifespan by maintaining the basal p38 MAPK/PMK-1 signaling (Figure 7C), our data show that mitochondrial stress promotes the longevity of *mbl-1(tm1563)* mutants in a partially PMK-1-dependent manner (Figure 7, A and B, Supplementary Figure S7, A and B and Tables S1 and S2). Although Western blot and qRT-PCR data (Figure 5, B–F, Supplementary Figure S4, Figure 6, C and D and Supplementary Table S8) indicate that *cox-5B* knockdown modulates the activity of p38 MAPK/PMK-1, it must be taken into account that p38 MAPK can also be activated by acetylation (Pillai *et al.* 2011), raising the possibility that mitochondrial stress promotes p38 MAPK/PMK-1 function through different mechanisms. Importantly, overactive p38 MAPK signaling is toxic and shortens lifespan (Cheesman *et al.* 2016), and therefore, it can be assumed that p38 MAPK signaling is only moderately activated during noninfectious conditions such as mitochondrial stress. Research in mammalian systems has demonstrated that p38 MAPK functions as a signaling hub that converts signals from multiple upstream factors into cellular phenotypes by modulating the function of several downstream effectors (Zarubin and Han 2005). Hence, it is possible that MBL-1 and mitochondrial function are among multiple factors fine-tuning the activity of p38 MAPK signaling, which determine its effect on gene expression, and consequently, on longevity. Since p38 MAPK/PMK-1 is only partially required for the increased *mbl-1(tm1563)* mutant lifespan upon mitochondrial stress (Figure 7, A and B, Supplementary Figure S7, A and B and Tables S1 and S2), there are also other mechanisms involved. Since DM1, which is associated with reduced MBNL1 activity, has been liked with mitochondrial changes (Ono *et al.* 1986; Gramegna *et al.* 2018), and MBNL1 is required for normal mitochondrial function in C2C12 cells (Yokoyama *et al.* 2020), it is likely that the loss of MBL-1 disrupts mitochondrial function [although mitochondrial genes are not enriched among differentially expressed genes in *mbl-1(tm1563)* mutants, Supplementary Table S4]. Therefore, mitochondrial perturbation-activated stress responses may restore the homeostasis in this organelle, thus leading to strikingly increased lifespan.

Taken together, these data elucidate an important mechanism of how the loss of DM1-associated muscleblind splicing factor affects lifespan. In terms of quality of life, it has been proposed that targeting aging may be a more efficient approach compared to targeting individual diseases (Kaeberlein *et al.* 2015). Therefore, since DM1 is a multisystemic disease with similarities to aging (Mateos-Aierdi *et al.* 2015; Meinke *et al.* 2018), this study suggests that targeting the aging process could provide a powerful complementary therapeutic approach for this severe disorder.

## Supplementary Material

iyab114_Supplementary_Data
