# Supplementary material for: Loss of muscleblind splicing factor shortens Caenorhabditis elegans lifespan by reducing the activity of p38 MAPK/PMK-1 and transcription factors ATF-7 and Nrf/SKN-1
Source: Genetics. 2021 Jul 22;219(2):iyab114. doi: 10.1093/genetics/iyab114 (PMC8633093; doi:10.1093/genetics/iyab114)
Supplement: iyab114_Supplementary_Data [file iyab114_supplementary_data.zip › iyab114-suppl_data/GENETICS-GENETICS-2021-304461-s08.docx]

**Supplemental Material legends**

**Fig S1. Overexpression of mCherry-tagged MBL-1**. (A) Screenshot from Ensembl genome browser representing *mbl-1* gene. Red arrows indicate isoforms expressed in *mbl-1* OE lines. Blue arrows indicate location of two promoter regions. (B) Day 1 adult animals expressing *mbl-1p::mbl-1::mCherry* under normal conditions (Ctrl, EV) and after acute oxidative stress (5 % H2O2 for 20 minutes, image taken after five hours recovery on EV). Abbreviations: n, neuron; v, vulva; s, spermatheca; i, intestine. Scale bar, 100 μm.

**Fig S2. MBL-1 activity decreases upon aging.** (A) Screenshot from WormBase presenting *unc-43* and *unc-104* genes. Arrows indicate exons whose exclusion was examined upon aging. (B-C) PCR-mediated splicing assay of *unc-43* and (D-E) *unc-104* genes. (B) and (D) show representative agarose gels and (C) and (E) agarose gel quantifications. In (C) and (E) bars represent the intensity of shorter PCR product (exon excluded) relative to longer PCR product (exon included) with error bars indicating mean ± s.d. of three biological replicates (**p < 0.01, two-way ANOVA with Tukey’s test). See S3 Table for agarose gel quantifications.

**Fig S3. Western blot repeats for figures 2C, 3A and 3C.** See S6 Table for Western blot quantifications.

**Fig S4. Western blot repeats for figures 3A, 5B and 5E.** See S6 Table for Western blot quantifications.

**Fig S5. Lifespan effects upon depletion of p38 MAPK components and key regulators.** (A) *tir-1* RNAi causes reduction in N2 lifespan but does not affect the lifespan of *mbl-1(tm1563)* mutants. (B) *mbl-1* RNAi shortens N2 lifespan but does not affect the short lifespan of *nsy-1(ag3)* and *sek-1(km4)* mutants. (C) *mek-1* RNAi does not affect N2 lifespan but shortens *mbl-1(tm1563)* mutant lifespan. (D) *vhp-1* RNAi initiated at day 1 adult-stage shortens both N2 and *mbl-1(tm1563)* mutant lifespan. See S1-S2 Tables for lifespan statistics.

**Fig S6. Short lifespan of mbl-1(tm1563) mutants is not due to the aberrant function of DAF-16.** (A) Expression of DAF-16 regulated genes in N2 and *mbl-1(tm1563)* mutants. Animals were grown on EV and collected for qRT-PCR analysis at day 2 adult stage. Bars represent mRNA levels relative to N2 with error bars indicating mean ± s.d. of three biological replicates, each with three technical replicates (**p < 0.01, unpaired Student’s *t*-test). See S8 Table for raw qRT-PCR data. (B) *daf-16* RNAi shortens both N2 (p < 0.01) and *mbl-1(tm1563)* mutant (p < 0.01) lifespan. (C) *daf-2* RNAi increases both N2 (p < 0.01) and *mbl-1(tm1563)* mutant (p < 0.01) lifespans. See S1-S2 Tables for lifespan statistics.

**Fig S7. Lifespan effects upon knockdown of mitochondrial electron transport chain subunit *nduf-6*.** (A-B) *nduf-6* RNAi-treated *mbl-1(tm1563)* mutants have extended longevity compared to *nduf-6* RNAi-treated *pmk-1(km25);mbl-1(tm1563)* mutants. (B) shows the sub-set of lifespan curves presented in (A). See S1-S2 Tables for lifespan statistics.

**S1 Table. Summary of *C. elegans* lifespan experiments.** S1 Table can be found from the Supporting information-file.

**S2 Table. Individual replicates of *C. elegans* lifespan experiments.** S2 Table can be found from the Supporting information-file.

**S3 Table. Agarose gel quantifications.**

**S4 Table. List of up- and downregulated genes in day 2 adult *mbl-1(tm1563)* mutants.**

**S5 Table. WormExp output for up- and downregulated genes in day 2 adult *mbl-1(tm1563)* mutants.**

**S6 Table. Western blot quantifications.**

**S7 Table. Alternative splicing analysis of RNA-seq data.**

**S8 Table. Raw qRT-PCR and *gst-4p::gfp* imaging data.**

**S9 Table. Oligonucleotide sequences used for qRT-PCR in this study.** S9 Table can be found from the Supporting information-file.

**S10 Table. Oligonucleotide sequences used for splicing assay in this study.** S10 Table can be found from the Supporting information-file.
