# Supplementary material for: Loss of muscleblind splicing factor shortens Caenorhabditis elegans lifespan by reducing the activity of p38 MAPK/PMK-1 and transcription factors ATF-7 and Nrf/SKN-1
Source: Genetics. 2021 Jul 22;219(2):iyab114. doi: 10.1093/genetics/iyab114 (PMC8633093; doi:10.1093/genetics/iyab114)
Supplement: iyab114_Supplementary_Data [file iyab114_supplementary_data.zip › iyab114-suppl_data/GENETICS-GENETICS-2021-304461-s09.docx]

**Supplemental Table 1**

**S1 Table. Summary of *C. elegans* lifespan experiments.**

| Genotype, RNAi and treatment | mean lifespan  ± SE (days) | variation  compared to  control (%) | *P*-values  against  control | N (trials) |
| --- | --- | --- | --- | --- |
| Figure 1A | | | | |
| N2, HT115 | 20 ± 0.27 |  |  | 166 (2) |
| *mbl-1(tm1563)*, HT115 | 16.4 ± 0.16 | - 18 | <10^-3^ | 169 (2) |
| N2, OP50 | 17.3 ± 0.28 | - 13.5 | <10^-3^ | 165 (2) |
| *mbl-1(tm1563)*, OP50 | 15.2 ± 0.16 | - 24  § - 12.1 | <10^-3^  § <10^-3^ | 167 (2) |
| Figure 1B | | | | |
| N2, EV RNAi | 20.4 ± 0.34 |  |  | 152 (2) |
| N2, *mbl-1* RNAi | 16.0 ± 0.18 | - 21.6 | <10^-3^ | 152 (2) |
| Figure 1C | | | | |
| N2, EV RNAi | 20.3 ± 0.35 |  |  | 166 (2) |
| *mbl-1* OE line 1, EV RNAi | 16.7 ± 0.29 | - 17.7 | <10^-3^ | 159 (2) |
| *mbl-1* OE line 2, EV RNAi | 16.4 ± 0.31 | - 19.2 | <10^-3^ | 162 (2) |
| Figure 2B | | | | |
| N2, PA14 | 2.2 ± 0.03 |  |  | 175 (2) |
| *mbl-1(tm1563)*, PA14 | 2.3 ± 0.03 | + 4 | 0.511 | 162 (2) |
| *pmk-1(km25)*, PA14 | 1.4 ± 0.03 | - 36 | <10^-3^ | 181 (2) |
| Figure 3G | | | | |
| N2, EV RNAi | 21.2 ± 0.3 |  |  | 170 (2) |
| N2, *pmk-1* RNAi | 17.3 ± 0.18 | - 18.4 | <10^-3^ | 168 (2) |
| *mbl-1(tm1563)*, EV RNAi | 15.8 ± 0.33 | - 25.5 | <10^-3^ | 152 (2) |
| *mbl-1(tm1563)*, *pmk-1* RNAi | 16.9 ± 0.2 | - 20  # + 6.5 | <10^-3^  # 0.605 | 158 (2) |
| Figure 4A | | | | |
| N2, EV RNAi | 22.9 ± 0.42 |  |  | 156 (2) |
| N2, *atf-7* RNAi | 19.7 ± 0.34 | - 14 | <10^-3^ | 161 (2) |
| *mbl-1(tm1563)*, EV RNAi | 18.1 ± 0.25 | - 21 | <10^-3^ | 162 (2) |
| *mbl-1(tm1563)*, *atf-7* RNAi | 17.2 ± 0.24 | - 24.9  # - 5 | <10^-3^  # 0.0291 | 168 (2) |
| Figure 4B | | | | |
| N2, EV RNAi | 21.4 ± 0.41 |  |  | 148 (2) |
| N2, *skn-1* RNAi | 18.4 ± 0.22 | - 14 | <10^-3^ | 158 (2) |
| *mbl-1(tm1563)*, EV RNAi | 16.8 ± 0.3 | - 21.5 | <10^-3^ | 158 (2) |
| *mbl-1(tm1563)*, *skn-1* RNAi | 16.5 ± 0.23 | - 22.9  # - 1.8 | <10^-3^  # 0.0151 | 161 (2) |
| Figure 5A | | | | |
| N2, EV RNAi | 21.6 ± 0.32 |  |  | 193 (2) |
| N2, *cox-5B* RNAi | 25.5 ± 0.29 | + 15.3 | <10^-3^ | 200 (2) |
| *mbl-1(tm1563)*, EV RNAi | 18.0 ± 0.23 | - 16.7 | <10^-3^ | 181 (2) |
| *mbl-1(tm1563)*, *cox-5B* RNAi | 26.1 ± 0.4 | + 17.2  # + 31  ¶ + 2.3 | <10^-3^  # <10^-3^  ¶ <10-3 | 185 (2) |
| Figure 7A-7B | | | | |
| N2, EV RNAi | 22.7 ± 0.39 |  |  | 150 (2) |
| N2, *cox5B* RNAi | 29.2 ± 0.38 | + 22.2  @ - 9 | <10^-3^  @ <10^-3^ | 153 (2) |
| *mbl-1(tm1563)*, EV RNAi | 17.6 ± 0.24 | - 22.5 | <10^-3^ | 142 (2) |
| *mbl-1(tm1563)*, *cox5B* RNAi | 32.1 ± 0.35 | + 29.3  # + 45.2 | <10^-3^  # <10^-3^ | 149 (2) |
| *pmk-1(km25)*, EV RNAi | 19.1 ± 0.36 | - 15.9 | <10^-3^ | 149 (2) |
| *pmk-1(km25)*, *cox-5B* RNAi | 26 ± 0.41 | + 12.7  **@** -19  ***** + 26.5 | <10^-3^  @ <10^-3^  * <10^-3^ | 152 (2) |
| *pmk-1(km25);mbl-1(tm1563)*, EV RNAi | 17.7 ± 0.28 | - 22 | <10^-3^ | 145 (2) |
| *pmk-1(km25);mbl-1(tm1563)*, *cox-5B* RNAi | 28.5 ± 0.42 | + 20.4  @ - 11.2  & + 37.9 | <10^-3^  @ <10^-3^  & <10^-3^ | 150 (2) |
| Supplemental figure 5A | | | | |
| N2, EV RNAi | 22.3 ± 0.45 |  |  | 148 (2) |
| N2, *tir-1* RNAi | 18.8 ± 0.35 | - 15.7 | <10^-3^ | 153 (2) |
| *mbl-1(tm1563)*, EV RNAi | 17.1 ± 0.26 | - 23.3 | <10^-3^ | 175 (2) |
| *mbl-1(tm1563)*, *tir-1* RNAi | 16.8 ± 0.27 | - 24.7  # - 1.8 | <10^-3^  # 0.345 | 159 (2) |
| Supplemental figure 5B | | | | |
| N2, EV RNAi | 20.5 ± 0.32 |  |  | 172 |
| N2, *mbl-1* RNAi | 17.4 ± 0.23 | - 15.1 | <10^-3^ | 177 |
| *nsy-1(ag3*), EV RNAi | 14.4 ± 0.19 | - 29.8 | <10^-3^ | 170 |
| *nsy-1(ag3*), *mbl-1* RNAi | 14.8 ± 0.18 | - 27.8  < + 2.7 | <10^-3^  < 0.213 | 178 |
| *sek-1(km4)*, EV RNAi | 14.2 ± 0.21 | - 30.7 | <10^-3^ | 171 |
| *sek-1(km4)*, *mbl-1* RNAi | 14.5 ± 0.2 | - 29.3  > + 2.1 | <10^-3^  > 0.699 | 176 |
| Supplemental figure 5C | | | | |
| N2, EV RNAi | 22.9 ± 0.4 |  |  | 157 (2) |
| N2, *mek-1* RNAi | 23 ± 0.44 | + 0.4 | 0.543 | 165 (2) |
| *mbl-1(tm1563)*, EV RNAi | 17.5 ± 0.24 | - 23.6 | <10^-3^ | 171 (2) |
| *mbl-1(tm1563)*, *mek-1* RNAi | 15.8 ± 0.24 | - 31  # - 9.7 | <10^-3^  # <10^-3^ | 165 (2) |
| Supplemental figure 5D | | | | |
| N2, EV RNAi | 22.5 ± 0.53 |  |  | 78 (1) |
| N2, *vhp-1* RNAi (from d1 adults) | 18.9 ± 0.43 | - 16 | <10^-3^ | 77 (1) |
| *mbl-1(tm1563)*, EV RNAi | 17.6 ± 0.3 | - 21.8 | <10^-3^ | 64 (1) |
| *mbl-1(tm1563)*, *vhp-1* RNAi (from d1 adults) | 16 ± 0.4 | - 28.9  # - 9.1 | <10^-3^  # 0.005 | 39 (1) |
| Supplemental figure 6B | | | | |
| N2, EV RNAi | 22.1 ± 0.35 |  |  | 156 (2) |
| N2, *daf-16* RNAi | 20.6 ± 0.33 | - 6.8 | <10^-3^ | 162 (2) |
| *mbl-1(tm1563)*, EV RNAi | 17.8 ± 0.27 | - 19.5 | <10^-3^ | 165 (2) |
| *mbl-1(tm1563)*, *daf-16* RNAi | 16.2 ± 0.24 | - 26.7  # - 9 | <10^-3^  # <10^-3^ | 166 (2) |
| Supplemental figure 6C | | | | |
| N2, EV RNAi | 23.5 ± 0.58 |  | <10^-3^ | 75 (1) |
| N2, *daf-2* RNAi | 34.5 ± 0.5 | + 31.9 | <10^-3^ | 79 (1) |
| *mbl-1(tm1563)*, EV RNAi | 18.1 ± 0.39 | - 23 | <10^-3^ | 81 (1) |
| *mbl-1(tm1563)*, *daf-2* RNAi | 26.9 ± 0.6 | + 12.6  # + 32.7 | <10^-3^  # <10^-3^ | 85 (1) |
| Supplemental figure 7A-7B | | | | |
| N2, EV RNAi | 20.4 ± 0.48 |  |  | 70 |
| N2, *nduf-6* RNAi | 24.2 ± 0.33 | + 15.7  @ + 4.1 | <10^-3^  @ 0.07 | 85 |
| *mbl-1(tm1563)*, EV RNAi | 16.1 ± 0.22 | - 21.1 | <10^-3^ | 77 |
| *mbl-1(tm1563)*, *nduf-6* RNAi | 23.2 ± 0.38 | + 12.1  # + 30.6 | 0.017 | 79 |
| *pmk-1(km25)*, EV RNAi | 18.1 ± 0.37 | - 11.3 | <10^-3^ | 85 |
| *pmk-1(km25)*, *nduf-6* RNAi | 20.4 ± 0.49 | 0  * + 11.3  @ - 12.1 | 0.3918  * <10^-3^  @ <10^-3^ | 83 |
| *pmk-1(km25);mbl-1(tm1563)*, EV RNAi | 16.4 ± 0.2 | - 19.6 | <10^-3^ | 78 |
| *pmk-1(km25);mbl-1(tm1563)*, *nduf-6* RNAi | 20.5 ± 0.38 | + 0.5  & + 20  @ - 11.6 | 0.0326  & <10^-3^  @ <10^-3^ | 80 |

§ Compared to N2, OP50 treatment

# Compared to *mbl-1(tm1563)*, EV RNAi treatment

¶ Compared to N2, *cox-5B* RNAi treatment

@ Compared to *mbl-1(tm1563)*, *cox-5B* or *nduf-6* RNAi treatment

* Compared to *pkm-1(km25)*, EV treatment

& Compared to *pkm-1(km25);mbl-1(tm1563)*, EV treatment

< Compared to *nsy-1(ag3)*, EV treatment

> Compared to *sek-1(km4)*, EV treatment
