# Supplementary material for: Loss of muscleblind splicing factor shortens Caenorhabditis elegans lifespan by reducing the activity of p38 MAPK/PMK-1 and transcription factors ATF-7 and Nrf/SKN-1
Source: Genetics. 2021 Jul 22;219(2):iyab114. doi: 10.1093/genetics/iyab114 (PMC8633093; doi:10.1093/genetics/iyab114)
Supplement: iyab114_Supplementary_Data [file iyab114_supplementary_data.zip › iyab114-suppl_data/GENETICS-GENETICS-2021-304461-s10.docx]

**Supplemental Table 2**

**S2 Table. Individual replicates of *C. elegans* lifespan experiments.**

| Genotype, RNAi and treatment | mean lifespan  ± SE (days) | variation  compared to  control (%) | *P*-values  against  control | N |
| --- | --- | --- | --- | --- |
| Figure 1A | | | | |
| N2, HT115, rep1 | 19.8 ± 0.31 |  |  | 86 |
| N2, OP50, rep1 | 16.4 ± 0.33 | - 17.2 | <10^-3^ | 87 |
| *mbl-1(tm1563)*, HT115, rep1 | 16.5 ± 0.18 | - 16.7 | <10^-3^ | 86 |
| *mbl-1(tm1563)*, OP50, rep1 | 15 ± 0.22 | - 24.2  § - 8.5 | <10^-3^  § <10^-3^ | 84 |
| N2, HT115, rep2 | 19.9 ± 0.41 |  |  | 80 |
| N2, OP50, rep2 | 18.2 ± 0.42 | - 8.5 | <10^-3^ | 78 |
| *mbl-1(tm1563)*, HT115, rep2 | 16.3 ± 0.27 | - 18.1 | <10^-3^ | 83 |
| *mbl-1(tm1563)*, OP50, rep2 | 15.3 ± 0.22 | - 23.1  § - 6.1 | <10^-3^  § <10^-3^ | 83 |
| Figure 1B | | | | |
| N2, EV RNAi, rep1 | 20.5 ± 0.48 |  |  | 76 |
| N2, *mbl-1* RNAi, rep1 | 16.0 ± 0.28 | - 22 | <10^-3^ | 75 |
| N2, EV RNAi, rep2 | 20.1 ± 0.45 |  |  | 76 |
| N2, *mbl-1* RNAi, rep2 | 16.0 ± 0.24 | - 20.4 | <10^-3^ | 77 |
| Figure 1C | | | | |
| N2, EV RNAi, rep1 | 21.1 ± 0.47 |  |  | 88 |
| *mbl-1* OE line 1, EV RNAi, rep1 | 17.8 ± 0.40 | - 15.6 | <10^-3^ | 83 |
| *mbl-1* OE line 2, EV RNAi, rep1 | 17.3 ± 0.43 | - 18 | <10^-3^ | 85 |
| N2, EV RNAi, rep2 | 19.4 ± 0.5 |  |  | 78 |
| *mbl-1* OE line 1, EV RNAi, rep2 | 15.4 ± 0.37 | - 20.6 | <10^-3^ | 76 |
| *mbl-1* OE line 2, EV RNAi, rep2 | 15.3 ± 0.41 | - 21.1 | <10^-3^ | 77 |
| Figure 2B | | | | |
| N2, PA14, rep1 | 2.2 ± 0.04 |  |  | 89 |
| *mbl-1(tm1563)*, PA14, rep1 | 2.2 ± 0.04 | 0 | 0.665 | 84 |
| *pmk-1(km25)*, PA14, rep1 | 1.4 ± 0.05 | - 36 | <10^-3^ | 92 |
| N2, PA14, rep2 | 2.2 ± 0.04 |  |  | 86 |
| *mbl-1(tm1563)*, PA14, rep2 | 2.2 ± 0.04 | 0 | 0.619 | 78 |
| *pmk-1(km25)*, PA14, rep2 | 1.4 ± 0.07 | - 36 | <10^-3^ | 89 |
| Figure 3G | | | | |
| N2, EV RNAi, rep1 | 19.3 ± 0.28 |  |  | 86 |
| N2, *pmk-1* RNAi, rep1 | 17.3 ± 0.19 | - 10.4 | <10^-3^ | 88 |
| *mbl-1(tm1563)*, EV RNAi, rep1 | 15.8 ± 0.22 | - 18.1 | <10^-3^ | 63 |
| *mbl-1(tm1563)*, *pmk-1* RNAi, rep1 | 16.1 ± 0.16 | - 16.6  # + 1.9 | <10^-3^  # 0.89 | 81 |
| N2, EV RNAi, rep2 | 21.2 ± 0.37 |  |  | 84 |
| N2, *pmk-1* RNAi, rep2 | 17.4± 0.29 | - 17.9 | <10^-3^ | 80 |
| *mbl-1(tm1563)*, EV RNAi, rep2 | 15.5 ± 0.57 | - 26.9 | <10^-3^ | 89 |
| *mbl-1(tm1563)*, *pmk-1* RNAi, rep2 | 17.6 ± 0.34 | - 17  # + 11.9 | <10^-3^  # 0.297 | 77 |
| Figure 4A | | | | |
| N2, EV RNAi, rep1 | 20.8 ± 0.42 |  |  | 73 |
| N2, *atf-7* RNAi, rep1 | 18.7 ± 0.35 | - 10.1 | <10^-3^ | 78 |
| *mbl-1(tm1563)*, EV RNAi, rep1 | 17.8 ± 0.27 | - 14.4 | <10^-3^ | 75 |
| *mbl-1(tm1563)*, *atf-7* RNAi, rep1 | 17.0 ± 0.28 | - 18.3  # - 4.5 | <10^-3^  # 0.17 | 82 |
| N2, EV RNAi, rep2 | 23.3 ± 0.59 |  |  | 83 |
| N2, *atf-7* RNAi, rep2 | 20.5 ± 0.55 | - 12 | <10^-3^ | 83 |
| *mbl-1(tm1563)*, EV RNAi, rep2 | 18.4 ± 0.4 | - 21 | <10^-3^ | 87 |
| *mbl-1(tm1563)*, *atf-7* RNAi, rep2 | 17.4 ± 0.38 | - 25.3  # - 5.4 | <10^-3^  # 0.137 | 86 |
| Figure 4B | | | | |
| N2, EV RNAi, rep1 | 21.8 ± 0.51 |  |  | 73 |
| N2, *skn-1* RNAi, rep1 | 18.9 ± 0.4 | - 13.3 | <10^-3^ | 75 |
| *mbl-1(tm1563)*, EV RNAi, rep1 | 16.8 ± 0.31 | - 22.9 | <10^-3^ | 72 |
| *mbl-1(tm1563)*, *skn-1* RNAi, rep1 | 16.7 ± 0.21 | - 23.4  # - 0.6 | <10^-3^  # 0.314 | 81 |
| N2, EV RNAi, rep2 | 20 ± 0.55 |  |  | 75 |
| N2, *skn-1* RNAi, rep2 | 18 ± 0.21 | - 10 | <10^-3^ | 83 |
| *mbl-1(tm1563)*, EV RNAi, rep2 | 16.6 ± 0.5 | - 17 | <10^-3^ | 86 |
| *mbl-1(tm1563)*, *skn-1* RNAi, rep2 | 16.3 ± 0.4 | - 18.5  # - 1.8 | <10^-3^  # 0.0137 | 80 |
| Figure 5A | | | | |
| N2, EV RNAi, rep1 | 21.6 ± 0.53 |  |  | 81 |
| N2, *cox-5B* RNAi, rep1 | 26.1 ± 0.48 | + 17.2 | <10^-3^ | 85 |
| *mbl-1(tm1563)*, EV RNAi, rep1 | 16.3 ± 0.26 | - 24.5 | <10^-3^ | 78 |
| *mbl-1(tm1563)*, *cox-5B* RNAi, rep1 | 26.7 ± 0.68 | + 19.1  # + 39  ¶ + 2.2 | <10^-3^  # <10^-3^  ¶ 0.021 | 80 |
| N2, EV RNAi, rep2 | 21.5 ± 0.39 |  |  | 112 |
| N2, *cox-5B* RNAi, rep2 | 25 ± 0.34 | + 14 | <10^-3^ | 115 |
| *mbl-1(tm1563)*, EV RNAi, rep2 | 19.2 ± 0.29 | - 10.7 | <10^-3^ | 103 |
| *mbl-1(tm1563)*, *cox-5B* RNAi, rep2 | 25.6 ± 0.45 | + 16  # + 25  ¶ + 2.3 | <10^-3^  # <10^-3^  ¶ 0.037 | 105 |
| Figure 7A-7B | | | | |
| N2, EV RNAi, rep1 | 22.5 ± 0.6 |  |  | 72 |
| N2, *cox5B* RNAi, rep1 | 30.6 ± 0.5 | + 26.5  @ - 6.9 | <10^-3^  @ 0.001 | 80 |
| *mbl-1(tm1563)*, EV RNAi, rep1 | 18.1 ± 0.33 | - 19.6 | <10^-3^ | 73 |
| *mbl-1(tm1563)*, *cox5B* RNAi, rep1 | 32.9 ± 0.44 | + 31.6  # + 45 | <10^-3^  # <10^-3^ | 75 |
| *pmk-1(km25)*, EV RNAi, rep1 | 18.8 ± 0.53 | - 16.4 | <10^-3^ | 79 |
| *pmk-1(km25)*, *cox-5B* RNAi, rep1 | 27.1 ± 0.53 | + 17  **@** - 17.6  *** + 30.6** | <10^-3^  @ <10^-3^  * <10^-3^ | 75 |
| *pmk-1(km25);mbl-1(tm1563)*, EV RNAi, rep1 | 18 ± 0.38 | - 20 | <10^-3^ | 74 |
| *pmk-1(km25);mbl-1(tm1563)*, *cox-5B* RNAi, rep1 | 28.2 ± 0.68 | + 20.2  @ - 14.3  & + 36.2 | <10^-3^  @ <10^-3^  & <10^-3^ | 74 |
| N2, EV RNAi, rep2 | 22.9 ± 0.49 |  |  | 78 |
| N2, *cox5B* RNAi, rep2 | 27.3 ± 0.44 | + 16.1  @ - 9 | <10^-3^  @ <10^-3^ | 73 |
| *mbl-1(tm1563)*, EV RNAi, rep2 | 17.1 ± 0.34 | - 25.3 | <10^-3^ | 69 |
| *mbl-1(tm1563)*, *cox5B* RNAi, rep2 | 30 ± 0.39 | + 23.7  # + 43 | <10^-3^  # <10^-3^ | 74 |
| *pmk-1(km25)*, EV RNAi, rep2 | 19.4 ± 0.5 | - 15.3 | <10^-3^ | 70 |
| *pmk-1(km25)*, *cox-5B* RNAi, rep2 | 24.8 ± 0.55 | + 7.6  **@** - 17.3  ***** + 21.8 | <10^-3^  @ <10^-3^  * <10^-3^ | 77 |
| *pmk-1(km25);mbl-1(tm1563)*, EV RNAi | 17.4 ± 0.4 | - 24 | <10^-3^ | 71 |
| *pmk-1(km25);mbl-1(tm1563)*, *cox-5B* RNAi, rep2 | 28.3 ± 0.43 | + 19.1  @ - 5.6  & + 38.5 | <10^-3^  @ <10^-3^  & <10^-3^ | 76 |
| Supplemental figure 5A | | | | |
| N2, EV RNAi, rep1 | 21.3 ± 0.67 |  |  | 65 |
| N2, *tir-1* RNAi, rep1 | 18.2 ± 0.52 | - 14.6 | <10^-3^ | 72 |
| *mbl-1(tm1563)*, EV RNAi, rep1 | 15.9 ± 0.28 | - 25.4 | <10^-3^ | 88 |
| *mbl-1(tm1563)*, *tir-1* RNAi, rep1 | 15.9 ± 0.37 | - 25.4  # 0 | <10^-3^  # 0.12 | 80 |
| N2, EV RNAi, rep2 | 22.3 ± 0.48 |  | <10^-3^ | 83 |
| N2, *tir-1* RNAi, rep2 | 19.4 ± 0.46 | - 13 | <10^-3^ | 81 |
| *mbl-1(tm1563)*, EV RNAi, rep2 | 18.4 ± 0.38 | - 17.5 | <10^-3^ | 87 |
| *mbl-1(tm1563)*, *tir-1* RNAi, rep2 | 17.7 ± 0.35 | - 20.6  # - 3.8 | <10^-3^  # 0.613 | 79 |
| Supplemental figure 5B | | | | |
| N2, EV RNAi, rep1 | 20.5 ± 0.45 |  |  | 87 |
| N2, *mbl-1* RNAi, rep1 | 17.7 ± 0.33 | - 13.7 | <10^-3^ | 89 |
| *nsy-1(ag3*), EV RNAi, rep1 | 14.7 ± 0.26 | - 28.3 | <10^-3^ | 85 |
| *nsy-1(ag3*), *mbl-1* RNAi rep1 | 15 ± 0.26 | - 26.8  < + 2 | <10^-3^  < 0.448 | 88 |
| *sek-1(km4)*, EV RNAi, rep1 | 14.3 ± 0.32 | - 30.2 | <10^-3^ | 85 |
| *sek-1(km4)*, *mbl-1* RNAi, rep1 | 14.8 ± 0.28 | - 27.8  > + 3.4 | <10^-3^  > 0.569 | 87 |
| N2, EV RNAi, rep2 | 20.4 ± 0.47 |  |  | 85 |
| N2, *mbl-1* RNAi, rep2 | 17.1 ± 0.32 | - 16.2 | <10^-3^ | 88 |
| *nsy-1(ag3*), EV RNAi, rep2 | 14.1 ± 0.27 | - 30.9 | <10^-3^ | 85 |
| *nsy-1(ag3*), *mbl-1* RNAi rep2 | 14.6 ± 0.25 | - 28.4  < + 3.4 | <10^-3^  < 0.301 | 90 |
| *sek-1(km4)*, EV RNAi, rep2 | 14.2 ± 0.29 | - 30.4 | <10^-3^ | 86 |
| *sek-1(km4)*, *mbl-1* RNAi, rep2 | 14.2 ± 0.28 | - 30.4  > 0 | <10^-3^  > 0.959 | 89 |
| Supplemental figure 5C | | | | |
| N2, EV RNAi, rep1 | 22.9 ± 0.59 |  |  | 71 |
| N2, *mek-1* RNAi, rep1 | 23.3 ± 0.58 | + 1.7 | 0.742 | 81 |
| *mbl-1(tm1563)*, EV RNAi, rep1 | 17.8 ± 0.33 | - 22.3 | <10^-3^ | 85 |
| *mbl-1(tm1563)*, *mek-1* RNAi, rep1 | 16.2 ± 0.37 | - 29.3  # - 9 | <10^-3^  # 0.022 | 81 |
| N2, EV RNAi, rep2 | 23 ± 0.54 |  |  | 86 |
| N2, *mek-1* RNAi, rep2 | 22.7 ± 0.65 | - 1.3 | 0.533 | 84 |
| *mbl-1(tm1563)*, EV RNAi, rep2 | 17.2 ± 0.34 | - 25.2 | <10^-3^ | 86 |
| *mbl-1(tm1563)*, *mek-1* RNAi, rep2 | 15.4 ± 0.31 | - 33  # - 10.5 | <10^-3^  # 0.0016 | 84 |
| Supplemental figure 5D | | | | |
| N2, EV RNAi | 22.5 ± 0.53 |  |  | 78 |
| N2, *vhp-1* RNAi (from d1 adults) | 18.9 ± 0.43 | - 16 | <10^-3^ | 77 |
| *mbl-1(tm1563)*, EV RNAi | 17.6 ± 0.3 | - 21.8 | <10^-3^ | 64 |
| *mbl-1(tm1563)*, *vhp-1* RNAi (from d1 adults) | 16 ± 0.4 | - 28.9  # - 9.1 | <10^-3^  # 0.005 | 39 |
| Supplemental figure 6B | | | | |
| N2, EV RNAi, rep1 | 22.7 ± 0.48 |  |  | 75 |
| N2, *daf-16* RNAi, rep1 | 21.6 ± 0.48 | - 4.8 | <10^-3^ | 78 |
| *mbl-1(tm1563)*, EV RNAi, rep1 | 18.1 ± 0.39 | - 20.3 | <10^-3^ | 81 |
| *mbl-1(tm1563)*, *daf-16* RNAi, rep1 | 17.1 ± 0.28 | - 24.7  # - 5.5 | <10^-3^  # <10^-3^ | 83 |
| N2, EV RNAi, rep2 | 21.4 ± 0.47 |  |  | 81 |
| N2, *daf-16* RNAi, rep2 | 19.6 ± 0.42 | - 8.4 | <10^-3^ | 84 |
| *mbl-1(tm1563)*, EV RNAi, rep2 | 17.4 ± 0.38 | - 18.7 | <10^-3^ | 84 |
| *mbl-1(tm1563)*, *daf-16* RNAi, rep2 | 15.4 ± 0.36 | - 28  # - 11.5 | <10^-3^  # <10^-3^ | 83 |
| Supplemental figure 6C | | | | |
| N2, EV RNAi | 23.5 ± 0.58 |  | <10^-3^ | 75 |
| N2, *daf-2* RNAi | 34.5 ± 0.5 | + 31.9 | <10^-3^ | 79 |
| *mbl-1(tm1563)*, EV RNAi | 18.1 ± 0.39 | - 23 | <10^-3^ | 81 |
| *mbl-1(tm1563)*, *daf-2* RNAi | 26.9 ± 0.6 | + 12.6  # + 32.7 | <10^-3^  # <10^-3^ | 85 |
| Supplemental figure 7A-7B | | | | |
| N2, EV RNAi | 20.4 ± 0.48 |  |  | 70 |
| N2, *nduf-6* RNAi | 24.2 ± 0.33 | + 15.7  @ + 4.1 | <10^-3^  @ 0.07 | 85 |
| *mbl-1(tm1563)*, EV RNAi | 16.1 ± 0.22 | - 21.1 | <10^-3^ | 77 |
| *mbl-1(tm1563)*, *nduf-6* RNAi | 23.2 ± 0.38 | + 12.1  # + 30.6 | 0.017 | 79 |
| *pmk-1(km25)*, EV RNAi | 18.1 ± 0.37 | - 11.3 | <10^-3^ | 85 |
| *pmk-1(km25)*, *nduf-6* RNAi | 20.4 ± 0.49 | 0  * + 11.3  @ - 12.1 | 0.3918  * <10^-3^  @ <10^-3^ | 83 |
| *pmk-1(km25);mbl-1(tm1563)*, EV RNAi | 16.4 ± 0.2 | - 19.6 | <10^-3^ | 78 |
| *pmk-1(km25);mbl-1(tm1563)*, *nduf-6* RNAi | 20.5 ± 0.38 | + 0.5  & + 20  @ - 11.6 | 0.0326  & <10^-3^  @ <10^-3^ | 80 |

§ Compared to N2, OP50 treatment

# Compared to *mbl-1(tm1563)*, EV RNAi treatment

¶ Compared to N2, *cox-5B* RNAi treatment

@ Compared to *mbl-1(tm1563)*, *cox-5B* RNAi treatment

* Compared to *pkm-1(km25)*, EV treatment

& Compared to *pkm-1(km25);mbl-1(tm1563)*, EV treatment

< Compared to *nsy-1(ag3)*, EV treatment

> Compared to *sek-1(km4)*, EV treatment
