# Supplementary material for: Loss of muscleblind splicing factor shortens Caenorhabditis elegans lifespan by reducing the activity of p38 MAPK/PMK-1 and transcription factors ATF-7 and Nrf/SKN-1
Source: Genetics. 2021 Jul 22;219(2):iyab114. doi: 10.1093/genetics/iyab114 (PMC8633093; doi:10.1093/genetics/iyab114)
Supplement: iyab114_Supplementary_Data [file iyab114_supplementary_data.zip › iyab114-suppl_data/GENETICS-GENETICS-2021-304461-s17.docx]

**Supplemental Table 9**

**S9 Table. Oligonucleotide sequences used for qRT-PCR in this study.**

| Gene | Forward (5’ 🡪 3’) | Reverse (5’ 🡪 3’) |
| --- | --- | --- |
| *cdc-42* | CTGCTGGACAGGAAGATTACG | CTCGGACATTCTCGAATGAAG |
| *pmp-3* | GTTCCCGTGTTCATCACTCAT | ACACCGTCGAGAAGCTGTAGA |
| *tir-1* | TGGAGTCAACCGGTCTCTTC | AGAGCATTGCAACTTCCACG |
| *nsy-1* | CACCAGAGCCAATCGTTTCC | TTGAGCACCACATGAAGCAC |
| *sek-1* | ATGGAGCAATGTTTCGCGAA | GACCGACAGAGCCATTTTCC |
| *pmk-1* | TGGATTGGCACGTCAAACTG | TGCAAGAATACACCCAACTGAC |
| *mek-1* | TTCCTGGTCTAGCATTCATGG | AGGGATTCGGTGAGAGAGGT |
| *vhp-1* | CTTCGATCTCGCCAAACTTC | TGGATGATGCACTTTTTGGA |
| *dod-24* | ATGGGTCAGAATCAGGTGGA | TCAGGCCCATCGTAACTTGT |
| *gst-10* | AAGAGATTGTGCAGACTGGAG | AGAACATGTCGAGGAAGGTTG |
| *C32H11.3* | CTTTCAAATGACGAAGTGGCG | GTCTGAATTAAACTCTGCGGC |
| *pcp-2* | GCTGGACCAATGGACATTTC | CCTTGCTCGTTGTCATTTGTAG |
| *B0024.4* | ATGGTCTCAAACTGAAGGCTC | AGAGAAAATTTCAACGACAGGAAAG |
| *F23F12.3* | CTACTTGCTCTGCCAATACTCC | CGTTCGACATATGAAATTCCCG |
| *C17H12.8* | CACTGTCGATTGCTCACTCC | TGTTGAGCTTGTGAAAGTGGA |
| *mul-1* | GGCTCAAACTACGTTGCTCA | GACGAATCCTTGCATGGTGG |
| *irg-4* | GAACCCATTAGCAATCTACCGT | GTTGAGCTTGTGACAGTGGG |
| *K08D8.5* | TCCGGGAAGTCGAATGAACA | CTGGAATTGAGAAGCCGACG |
| *C29F3.7* | CAAGACTGTGACGGCATTGT | GTTTTAGTGACCCCGCCATC |
| *catp-3* | CGACGTTGGAGTTGCTATGG | GTTCAGGCCACATGTGAGTC |
| *T25C12.3* | ACGCTGCTGATGAGACATCT | AGATCATTTGGCCCGTCTGA |
| *grd-3* | ACATGCCCATCACTCAAGGA | GCCTGAACGATTGGTCCAAG |
| *amt-1* | CGGGTATTGGGCTATCGGAT | CCGGAAACAATAGTAGCGGC |
