# Supplementary material for: Loss of muscleblind splicing factor shortens Caenorhabditis elegans lifespan by reducing the activity of p38 MAPK/PMK-1 and transcription factors ATF-7 and Nrf/SKN-1
Source: Genetics. 2021 Jul 22;219(2):iyab114. doi: 10.1093/genetics/iyab114 (PMC8633093; doi:10.1093/genetics/iyab114)
Supplement: iyab114_Supplementary_Data [file iyab114_supplementary_data.zip › iyab114-suppl_data/GENETICS-GENETICS-2021-304461-s18.docx]

**Supplemental Table 10**

**S10 Table. Oligonucleotide sequences used for splicing assay in this study.**

| Gene | Forward (5’ 🡪 3’) | Reverse (5’ 🡪 3’) |
| --- | --- | --- |
| *unc-43* | GACCACCATCGACAATCAAGG | CAACAACGTCTGAGTCACCC |
| *unc-104* | TGCCACCTGATCTATTGCCA | AACGAATGCTCTTCCAACCA |
